# Investigating Rates of Hunting and Survival in Declining European Lapwing Populations

**DOI:** 10.1371/journal.pone.0163850

**Published:** 2016-09-29

**Authors:** Guillaume Souchay, Michael Schaub

**Affiliations:** Swiss Ornithological Institute, Sempach, Switzerland; Oregon State University, UNITED STATES

## Abstract

Understanding effects of harvest on population dynamics is of major interest, especially for declining species. European lapwing *Vanellus vanellus* populations increased from the 1960s until the 1980s and declined strongly thereafter. About 400,000 lapwings are harvested annually and it is thus of high conservation relevance to assess whether hunting was a main cause for the observed changes in lapwing population trends. We developed a multi-event cause-specific mortality model which we applied to a long-term ring-recovery data set (1960–2010) of > 360,000 records to estimate survival and cause-specific mortalities. We found no temporal change in survival over the last 50 years for first-year (FY) and older birds (after first-year; AFY) originating from different ringing areas. Mean survival was high, around 0.60 and 0.80 for FY and AFY individuals, respectively. The proportion of total mortality due to hunting was <0.10 over the study period and the estimated proportion of harvested individuals (kill rate) was <0.05 in each year. Our result of constant survival indicates that demographic processes other than survival were responsible for the pronounced change in lapwing population trends in the 1980s. Our findings lend support to the hypothesis that hunting was not a significant contributor to the large-scale decline of lapwing populations. To halt the ongoing decline of European lapwing populations management should focus on life history stages other than survival (e.g. productivity). Further analyses are required to investigate the contribution of other demographic rates to the decline of lapwings and to identify the most efficient conservation actions.

## Introduction

Unmanaged exploitation of wildlife populations through hunting and fishing has affected them negatively [1]. Yet, if harvest is managed to be sustainable, populations should be unaffected in the long term. Sustainable harvest is defined as the level of exploitation ensuring that the total mortality (hunting and natural mortality combined) does not exceed recruitment [2]. The impact of hunting mortality can be placed on a continuum from: hunting acting as an additional source of mortality thereby increasing total mortality (i.e. totally additive) or total mortality remains constant despite fluctuation in hunting mortality (i.e. totally compensatory) [3]. Accurate estimates of hunting mortality and its effect on demographic parameters are needed for a sustainable harvest management [4]. However, reliable monitoring of survival and hunting mortality is lacking in Europe for most harvested species [2].

The lack of basic demographic information is especially problematic for harvested species that are declining such as the Northern lapwing (*Vanellus vanellus*; lapwing hereafter). Lapwings showed an increasing population trend during most of the 20^th^ century but have declined by 50% since the 1980s (see Appendix 1 of [5] for a review of changes in breeding populations during the last century). The lapwing is classified as Near Threatened (NT) on the Global Red List [6] and as Vulnerable (VU) on the recent European Red List of Birds [7]. The main reason for the population decline is presumably insufficient productivity due to agricultural intensification on breeding areas (see Fig 2 in [8–10]). However, it has been hypothesized that hunting may contribute to the observed changes in population trends given that about 400,000 individuals are harvested annually [11]. Lapwings are hunted in several European (i.e. France, Greece, Italy, Malta and Spain [11]) and North African countries, but it is unknown whether hunting is sustainable. Even the hunting bag (i.e. the number of individuals harvested per hunter) is not recorded accurately in most countries. The management plan of the European Union identified this lack of knowledge and urged for collecting proper hunting statistics and for a scientific assessment of lapwing hunting [11, 12].

Here, we estimated survival probabilities of lapwings from several European breeding populations over the last 50 years. Our goal was to assess whether hunting contributed to the marked change in lapwing population trends using ring-recovery data. These data are collected by national bird ringing schemes and stored in a central data base maintained by EURING [13](http://www.euring.org/edb/). Besides date and site information, the EURING databank (EDB) records the presumed mortality cause of a recovered ringed bird, which potentially allows the estimation of cause-specific mortality probabilities. However, a direct estimate of cause-specific mortality rates based on the frequency can be misleading because of imperfect detection or recovery probabilities that may be confounded with causes of death [14]. Schaub and Pradel [14] developed a multistate model that overcomes this difficulty and provides unbiased estimates of cause-specific mortality rates. The model assumes that the cause of death is known for all recovered individuals, but unfortunately, a substantial amount of recoveries of lapwings is recorded without information about the cause of death. We extended the Schaub and Pradel model [14] to account for the uncertainty in classifying cause of death. To facilitate comparison with other studies, we also derived an estimate of the kill rate (i.e. the probability that a lapwing died due to hunting in a given year). We investigated temporal and spatial patterns of survival, harvest and kill rates over 50 years. We would expect a decline in survival since the 1980s if it were the main reason for the change in lapwing population trends. We expected the proportion of mortality due to hunting to vary according to regionally specific hunting regulations. We discuss the potential impact of hunting on lapwing survival and its role (if any) for the current decline of lapwing populations.

## Methods

### EURING databank

We used lapwing ringing data from European breeding areas (North-western Europe: Denmark, Germany, Netherlands; Fennoscandia: Norway, Sweden, Finland; British Isles: England, Wales, Scotland, Ireland, Northern Ireland; see also Table A in S1 File for a complete list of contributing ringing schemes) and the subsequent recoveries across the whole of Europe and Northern Africa (Fig A in S1 File). Ringing and recovery information for each recovered lapwing was obtained from the EDB while the annual ringing totals were obtained from the national ringing schemes. The national ringing schemes were also asked to update the recovery data if needed. Because we were interested to study temporal variation in survival and in the proportion of harvested individuals, we used data from 1960–2010 (50 years).

We included only lapwings that were ringed as chicks (age 1 in the EDB) to ensure that the origin and age of all birds were clearly defined. Some of the ringed lapwings were recaptured or resighted alive, but due to their low number we excluded these encounters. Our analyses were based on 361,793 ringed chicks of which 6,209 were recovered dead (Table 1). Dead recoveries were typically found by the members of the public (e.g. hunters, farmers, bird watchers), who reported location, date and often also a presumed cause of death to national ringing schemes. We focused on the following causes of death: (i) unknown cause of death (EURING code for circumstances “00”, “01”, “03” and “99”, *N* = 1707), (ii) harvested (circumstances “10” to “19”, *N* = 2805), (iii) other causes (*N* = 1697). We used program R [15] and the package *Birdring* [16] for handling the recovery data.

**Table 1:** Summary of the numbers of lapwings ringed as chick in each country and subsequently recovered by areas. Recovery areas: BI (British Isles), NE (Northern Europe), SC (Fennoscandia) and WM (Western Mediterranean).

Area / Country	Years of ringing (n)	Numbers of ringed individuals	Numbers recovered per area				
			Total	BI	NE	SC	WM
			(% number ringed)	(% percentage of total recovered)			
*North-western Europe*							
Denmark	1969–2009 (41)	5,061	133 (2.6)	1 (0.8)	84 (63.2)	2 (1.5)	46 (34.6)
Germany	1976–2009 (33)	17,119	191 (1.1)	3 (1.6)	47 (24.6)	0 (0.0)	141 (73.8)
Netherlands	1960–2009 (50)	127,155	3,380 (2.6)	36 (1.1)	1,427 (42.2)	2 (0.1)	1,918 (56.7)
*British Isles*							
British Isles	1960–2009 (50)	155,520	1,401 (0.9)	1,110 (79.2)	11 (0.8)	5 (0.4)	275 (19.6)
*Fennoscandia*							
Finland	1960–2009 (50)	34,528	680 (2.0)	9 (1.3)	46 (6.8)	207 (30.4)	418 (61.5)
Norway	1960–2009 (50)	12,945	202 (1.6)	18 (8.9)	15 (7.4)	115 (56.9)	54 (26.7)
Sweden	1969–2009 (41)	9,465	222 (2.3)	5 (2.2)	23 (10.4)	82 (36.9)	112 (50.5)
*Total*		361,793	6,209 (1.7)	1,182 (19.0)	1,653 (26.6)	413 (6.7)	2,964 (47.7)

The recoveries were classified according to four regions where they were found, because recovery probabilities potentially differ geographically [17]. We distinguished between recoveries stemming from the British Isles (Channel Islands, Ireland, United Kingdom) plus the Faroes (named “BI” hereafter), Northwestern Europe (Belgium, Denmark, Germany, The Netherlands [NW]), Fennoscandia (Finland, Norway, Sweden [SC]) and countries around the Western Mediterranean Sea (Algeria, France, Italy, Malta, Morocco, Portugal and Spain, [WM]).

### Multievent cause-specific mortality model

Schaub and Pradel [14] developed a multistate capture-recapture model with which survival probabilities, proportions of mortality causes and cause-specific recovery probabilities can be estimated with the data as described above. A key feature of the model is that it allows the recovery probabilities to differ between the causes of death. However, the model assumes that the cause of death of all recovered individuals is known, which is a difficult assumption to fulfill. Schaub and Pradel [14] classified all recovered animals whose mortality cause was unknown to that cause of death that was not the focus (i.e. other causes) to mitigate this problem. However, depending on how the unknown causes of death are distributed among all causes of death, this may introduce bias in the estimated proportion of mortality causes. Therefore, we extended the cause-specific mortality model of Schaub and Pradel [14] to a multievent model [18] to account for uncertainty in the recorded causes of death of the recovered individuals.

Multievent models are efficient to estimate transition probabilities between imperfectly observed states [18]. These hierarchical models consist of two processes; the first describes the transition among biological states following a first order Markov Chain while the second process describes the observations (data) given the underlying biological states. The considered biological states were alive (A), newly dead due to hunting (NDh), newly dead due to another cause than hunting (NDo) and dead (D), each for the three geographical regions NW, SC, and BI where the lapwings hatched. The inclusion of the newly dead states was necessary to ensure that individuals found dead can only be attributed to one recovery period and is common in multistate models with dead individuals [19]. The state transition matrix was

$${\begin{array}{cc} & \;A\;NDh\;NDo\;D\;\\ \begin{array}{c} A\\ NDh\\ NDo\\ D \end{array} & \left[\begin{array}{cccc} S & \left(1-S\right)\alpha & \left(1-S\right)\left(1-\alpha \right) & 0\\ 0 & 0 & 0 & 1\\ 0 & 0 & 0 & 1\\ 0 & 0 & 0 & 1 \end{array}\right] \end{array}}_{t,a,reg}$$

where *S* is the annual survival probability and α the proportion of individuals that died due to hunting among those that died in a given year. All parameters in the matrix could vary depending on year *t*, age-class (*a*, first-year [FY] vs. after first-year [AFY]) and region of ringing (*reg*).

The observation matrix linked the four biological with the 14 observation states. 12 observation states were given by the combination of three mortality causes (“unknown”, “harvested”, “other than hunting”) and four recovery areas (coded 2–13). The observation states were completed by the states “not encountered” (coded 0) for individuals that died but were not recovered and “captured” for the initial capture of all individuals (coded 1). A list with a detailed description of the 14 observed states is given in S2 File. The observation matrix was the following:

$$\begin{array}{cc} & \;0\;1\;2\;3\;4\;5\;6\;7\;8\;9\;10\;11\;12\;13\;\\ \begin{array}{c} A\\ NDh\\ NDo\\ D \end{array} & {\left[\begin{array}{cccccccccccccc} 1 & 0 & 0 & 0 & 0 & 0 & 0 & 0 & 0 & 0 & 0 & 0 & 0 & 0\\ 1-\sum r_{h}^{i} & 0 & r_{h}^{BI}\delta_{h} & r_{h}^{NE}\delta_{h} & r_{h}^{SC}\delta_{h} & r_{h}^{WM}\delta_{h} & 0 & 0 & 0 & 0 & r_{h}^{BI}(1-\delta_{h}) & r_{h}^{NE}(1-\delta_{h}) & r_{h}^{SC}(1-\delta_{h}) & r_{h}^{WM}(1-\delta_{h})\\ 1-\sum r_{o}^{i} & 0 & 0 & 0 & 0 & 0 & r_{o}^{BI}\delta_{o} & r_{o}^{NE}\delta_{o} & r_{o}^{SC}\delta_{o} & r_{o}^{WM}\delta_{o} & r_{o}^{BI}\left(1-\delta_{o}\right) & r_{o}^{NE}(1-\delta_{o}) & r_{o}^{SC}\left(1-\delta_{o}\right) & r_{o}^{SC}\left(1-\delta_{o}\right)\\ 1 & 0 & 0 & 0 & 0 & 0 & 0 & 0 & 0 & 0 & 0 & 0 & 0 & 0 \end{array}\right]}_{t} \end{array}$$

where $r_{h}^{i}$ is the probability that an individual that was newly dead due to hunting was found in area *i* and its ring was recorded (i.e. recovery probability conditional on death due to hunting), $r_{o}^{i}$ is the probability that an individual that was newly dead due to another cause than hunting in region *i* was found and its ring was recorded, δ_*h*_ and δ_*o*_ are the probabilities that an individual that died due to hunting and due to another cause, respectively, were assigned to the correct cause of mortality. The assignment probabilities were assumed to be the same across all regions for sake of simplicity, while the cause-specific recovery probabilities were allowed to vary regionally.

### Main effects and model selection

The multievent cause-specific mortality model formulated above contains many parameters, which may not all be separately identifiable due to the model structure and sparse data. To minimize this potential problem, we constructed models with as few parameters as possible. We estimated probabilities of mortality proportions (α) and of survival (*S*) over 3- and 5-years periods, respectively. Because FY and AFY lapwings likely differ in their vulnerability to different sources of mortality, we also included an age effect (*a*, FY vs. AFY) in survival and in mortality proportions in our initial model. We assumed that the cause-specific recovery and the assignment probabilities were independent of age because these parameters are more related to the behavior of the reporter rather than to characteristic of the bird. Hunting legislation differs between regions. We therefore pooled recovery areas for the recovery rate conditional on hunting (*law* group effect: WM *vs* other areas), that is, recovery rate of harvested lapwings (*r*_*h*_) was supposed to differ between the Western Mediterranean Sea region and all others. The recovery probability of lapwings that died due to other reasons (*r*_*o*_) and the two assignment probabilities (δ_h_, δ_o_) were assumed to be constant over time and the same across all regions. Thus, our initial model (M1) was:

$$S_{\left[Reg*a*y5\right]},\alpha_{\left[Reg*a*y3\right]},r_{h\left[law*y5\right]},r_{o},\delta_{h},\;\delta_{o}.$$

For model selection, we focused first on cause-specific recovery probabilities while retaining full effect on the other parameters, then on survival and finally on the mortality cause probabilities [20]: (i) we restricted our investigation in cause-specific recovery probabilities to whether the temporal variation could be reduced to a linear trend over the last 50 years (model M2); (ii) we investigated the effects of age, region and time on survival probabilities (models M3-6); and then (iii) we looked at the effects of region and age on the proportion of hunting mortality (models M7-9). The proportion of hunting mortality (α) was time-dependent in each model, because α is confounded with the recovery probabilities and thus not separately estimable when it is constant over time [14]. Finally, we also tested if temporal variation in survival and proportions of mortality causes could be reduced to linear temporal trends (models M10 and M11). Model selection and parameter estimation were performed using program E-SURGE [21] (see S2 File for the implementation of the model in E-SURGE). Model selection relied on the Akaike’s Information Criterion (AIC [22]). All parameter estimates are given as mean ± SE.

Ring-recovery models with age-dependent survival and age-independent recovery probabilities are fully identifiable even when using recoveries from juveniles only [23]. However, more complex multistate models may be parameter redundant and thus produce biased estimates [24]. An extensive simulation study has shown that probabilities of mortality cause (α) and of cause-specific recovery (*r*) from the cause-specific mortality model can be biased [14] when the temporal variability of α gets too low, but that survival is always estimated without bias and thus robust [14, 25, 26]. We therefore had to evaluate whether estimates of α were reliable. To investigate parameter identifiability in the fitted multievent cause-specific mortality models, we used the diagnostic tool implemented in E-SURGE which is based on a hybrid symbolic-numerical method [27].

### Kill rates

The kill rate (*k*) is the probability that a lapwing is killed by a hunter in a given year [28]. It could directly be calculated as *k* = (1-*S*)α based on the estimates originating from the multievent cause-specific mortality model. However, we used a more classical approach to estimate *k* because the direct calculation has to date neither been investigated nor tested.

Following Anderson and Burnham [28] kill rates can be calculated from survival and recovery probabilities. This method was typically applied to birds that produced recoveries from hunting only. It must take into account the retrieval rate (*c*, the probability that a shot individual is retrieved by the hunter). The kill rate is calculated as:

k=f(λ*c), (1)

where λ is the reporting rate, i.e. the probability that the ring of a harvested individual is retrieved and reported, and *f*, the recovery rate, the probability that an individual is shot, retrieved and its ring reported by a hunter. The latter is calculated from the hunting recovery rate (ρ_*h*_), and the survival probability [19]:

$$f=\left(1-S\right)\rho_{h},$$ (2)

When Eq 2 is inserted in Eq 1 the kill rate is estimated as:

k=(1−S)ρhλ*c, (3)

We estimated kill rates for each ringing region, age class and time period using the corresponding probabilities of hunting recovery (ρ_*h*_) and survival (*S*). These parameters were obtained from a ring-recovery model accounting for uncertainty in assignment process (i.e. a multievent Seber model—see S3 File for a brief description).

Retrieval (*c*) and reporting rates (λ) were derived from the literature and assumed to be constant over region, age and time. Because no retrieval and reporting estimates are available for any bird species harvested in Europe [2, 29], we compiled estimates from the mourning dove (*Zenaida macroura*), the most similar species with available estimates (Tables A and B in S4 File). We have chosen a value of *c* = 0.75 ± 0.05 for the retrieval rate. For the reporting rate we used λ = 0.35 ± 0.05 during the first 30 years and λ = 0.10 ± 0.05 for the last 20 years. Different values were used, because reporting rates were likely to have declined due to the complete interruption of the recovery data processing by the French ringing center from 1992 to 2002 and the on-going slow data processing since 2002 (B. Trolliet, *pers*. *com*). We computed variance and standard errors of kill rate using the delta method [30].

## Results

### Model selection

Besides the most complex model, we fitted ten simpler models that included specific constraints (equality among age classes or areas, temporal trends). The global model (M1) received the strongest support by the data (Table 2). Models including trends in survival or in the proportion of hunting mortality were not supported by the data (ΔAIC ≥ 500). Age structure and geographic variation in survival and cause-specific mortality were evident (ΔAIC >15).

**Table 2:** Model selection results of survival and source of mortality of lapwings ringed as chicks and subsequently recovered from 1960 to 2010.

Model	*S*	α	*r*_*h*_	*r*_*o*_	δ_*h*_	δ_*h*_	NP	Deviance	ΔAIC
M1	Area*y5*a	Area*a*y3	law*y5	*y5*	-	-	194	107688.7	0.0
M9	Area*y5*a	*Area[NE = SC*,BI]*y3*a	law*y5	*y5*	-	-	160	107772.6	15.9
M6	*Area[NE = SC*,BI]*y5*a	Area*a*y3	law*y5	*y5*	-	-	174	107750.7	22.0
M7	Area*y5*a	Area*y3	law*y5	*y5*	-	-	143	107870.3	79.5
M3	y5*a	Area*a*y3	law*y5	*y5*	-	-	154	107953.6	184.9
M4	Area*y5	Area*a*y3	law*y5	*y5*	-	-	164	108276.4	527.6
M10	Area*a*T	Area*a*y3	law*y5	*y5*	-	-	146	108380.0	595.3
M8	Area*y5*a	a*y3	law*y5	*y5*	-	-	126	108732.3	907.6
M5	Area*a	Area*a*y3	law*y5	*y5*	-	-	140	110150.4	2353.7
M2	Area*y5*a	Area*a*y3	law*T	*T*	-	-	170	112619.2	4882.4
M11	Area*y5*a	Area*a*T	law*y5	*y5*	-	-	104	115706.4	7837.7

For each model, we give the number of estimated parameters (NP), the deviance and the difference in Akaike’s Information Criterion between each model and the best model (ΔAIC). The initial model was M1. Model notation: *Area*: geographic area of ringing with 3 main areas (NE = Northern Europe, SC = Scandinavia, BI = British Isles); *a*: age effect with 2 levels (first-year vs. after-first-year); *law*: recovery area effect to reflect differences in hunting legislations and traditions across recovery areas; *T*: temporal trend; *y3*: time effect pooling 3 consecutive years; *y5*: time effect pooling 5 consecutive years; - constant.

### Parameter estimates

The parameters of all fitted models were identifiable based on the diagnostic tool implemented in E-SURGE. However, the point estimates of all parameters (survival, cause-specific mortality and recovery) from the years 2000 to 2010 were either 1 or 0, indicating problems with estimation. As the models were intrinsically identifiable (as checked by E-SURGE), these estimation problems resulted from sparse data. Indeed, the number of recoveries was much lower in the period 2000–2010 compared to the previous periods (~150/year on average from 1960–2000; ~50/year on average from 2000–2010). We therefore based our inference only on parameter estimates from 1960 to 2000 (but see Fig A in S5 File for the complete results).

The annual recovery probabilities of harvested individuals in the Western Mediterranean were high (range: 0.88–0.92) in the first decades of our study but decreased strongly during the 1980s (mean = 0.08 ± 0.02) (Fig B in S5 File). In the other regions recovery probabilities of harvested individuals were lower (range: 0.00–0.04). Annual recovery probabilities of individuals that died from other causes were similar (range: 0.00–0.04). The probability that a lapwing that died due to hunting was assigned to the correct cause of mortality was close to one (δ_h_ = 0.98 ± 0.01).

**Fig 1:**
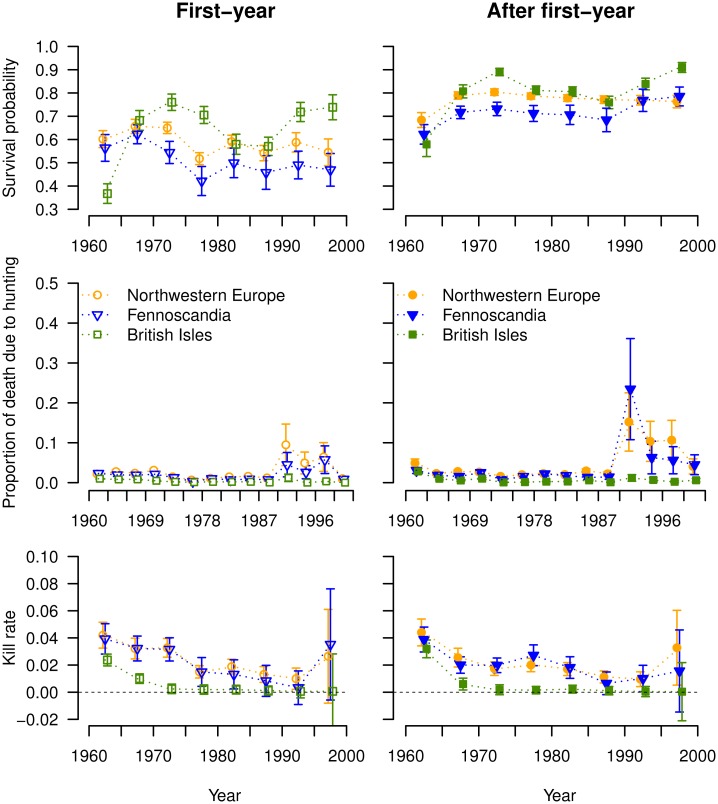
Estimates (± SE) of annual survival, proportion of mortality and kill rate probabilities of lapwings from 1960 to 2000 by area of ringing. Circles, triangles and squares represent North-western Europe, Fennoscandia and British Isles respectively. First row: survival probability, second row: hunting mortality, third row: kill rate. Left column: first-year individuals, Right column: after first-year individuals.

The annual survival probabilities varied with age, time and region. Annual survival of FY lapwings varied between 0.37 and 0.76, with the overall highest values for individuals originating from BI (${\bar{S}}_{BI}=0.64\pm 0.05$), intermediate values for NW lapwings (${\bar{S}}_{NW}=0.59\pm 0.02$) and lowest values for SC lapwings (${\bar{S}}_{SC}=0.51\pm 0.02$, Fig 1A). Annual survival of AFY lapwings seemed to have slightly increased over time in all areas (range for period 1960–1965: 0.58–0.68, range for period 1995–2000: 0.76–0.90), even though the model with linear time trends in survival was not well supported by the data (Table 2). Average AFY survival was again highest for BI lapwings (${\bar{S}}_{BI}=0.80\pm 0.04$), intermediate for NW lapwings (${\bar{S}}_{NW}=0.77\pm 0.01$) and lowest for SC lapwings (${\bar{S}}_{SC}=0.72\pm 0.02$, Fig 1B).

The proportion of death due to hunting (α) was relatively constant from 1960 to 1990 (Fig 1C and 1D), and was consistently higher for AFY than for FY individuals. In agreement with our expectations, the proportion was lowest for lapwings originating from BI. From 1990 onwards, the proportion of death due to hunting became highly variable over time and much elevated (but < 0.25) in all regions but BI, yet the estimation errors also increased.

### Kill rate

Based on the estimated survival and recovery probabilities from the multievent Seber model (Table A in S7 File), the kill rates of FY and AFY lapwings were low (< 0.05) and varied little over time from 1960 to 2000 (Fig 1E and 1F), even during the decline of the lapwing populations (from 1980 onwards). Mean kill rates were lower for lapwings from BI (0.005 ± 0.003 and 0.006 ± 0.004 for FY and AFY birds, respectively) than for lapwings originating from SC and NW (FY: 0.022 ± 0.005 and 0.023 ± 0.004, respectively; AFY: 0.019 ± 0.004 and 0.022 ± 0.004 respectively).

## Discussion

We investigated the impact of hunting on survival probabilities of lapwings from several declining European breeding populations over the last 50 years using ring recovery data. We found no decreasing trends in survival over time and a low hunting mortality that tended to decline. Our result of consistent survival probability indicates that other demographic processes than survival were responsible for the pronounced change in lapwing population trends in the 1980s. Our findings suggest that hunting was not a significant contributor to the large-scale decline of lapwing populations.

### Accouting for uncertainty in cause-specific mortality models

We extended the cause-specific mortality model of Schaub and Pradel [14] to account for recoveries associated with unknown causes of death. Formerly, such data were either removed or assigned to the mortality cause that was not the focus. Both approaches can result in biased estimates and increase uncertainty in inference (Figs A and B in S6 File). The extension of the cause-specific mortality model to the multievent framework allows the use of all data and to reliably estimate survival and proportions of cause-specific mortality.

Despite its appeal, some challenges for inference may arise when cause-specific mortality models are used. Schaub [25] pointed out that parameters related to the mortality cause (α, *r*) may not be identifiable when the temporal variability of α is too low (< 0.05). These estimation problems occur because a nested model with time-constant α is parameter redundant. This is known as near singularity [31] and may not be detected in E-SURGE.

To evaluate the performance of the multievent cause-specific mortality model we ran a few simulations based on Schaub [25]. We found that survival probabilities were always unbiased and the temporal patterns of the other parameters were estimated correctly, although they could be biased slightly. These results are consistent with the findings from the original cause-specific mortality model [25]. We also compared our survival estimates with estimates originating from a multievent Seber model [32], i.e. a ring-recovery model that used all ring recoveries with cause-specific recovery rates and accounting for uncertainty in assignment (S3 File). Such a model is known to produce unbiased estimates of survival and recovery [23]. Estimates of survival originating from our multievent cause-specific mortality model and from the multievent Seber model were similar (Fig A in S7 file). We also compared kill rates derived from our multievent cause specific mortality model and a multievent Seber model. We found that the potential bias in the kill rates from the multievent cause-specific mortality model is likely to be small (Fig B in S7 File) and our results are thus reliable.

The multievent cause-specific mortality model allows the estimation of survival and cause-specific mortality while taking into account uncertainty in the recovery process. It can be applied to investigate the relationship between specific causes of death and survival, provided that information about causes of death is available. Such information is recorded in the EURING databank of recovered European birds [13] and thus the model could be applied to study the impact of specific mortality causes on survival in various bird species.

### Spatial and temporal patterns in survival

Populations of long-lived species are typically sensitive to changes in AFY survival [33, 34], but AFY survival often exhibits less temporal variation than other demographic parameters [35]. Consistently, AFY survival seemed to exhibit less temporal variation than FY survival in each population considered here. FY survival of continental lapwings dropped slightly during the late 1970s and there was a decline in the early 1980s for British Isles populations. A likely explanation for these declines are severe winters [36]. FY survival increased from the 1990s for the British but not for the other populations. Similar temporal patterns in survival were found in previous studies for the British population [10, 37].

Survival of both age classes showed the same spatial patterns, it was lowest for lapwings originating from Fennoscandia, intermediate for Northwestern and highest for British lapwings. Several hypotheses might explain this gradient. Differential migration behavior results in different length of migratory flights, different wintering areas and differences in physiological costs. Most British lapwings are resident to the British Isles throughout the year and thus cover small migration distances and may benefit from "short-stopping" their migration [38] and avoiding the physiological costs and mortality risks of migration [39]. Close to 80% of lapwings ringed on the British Isles were recovered there, while only 20% of the recoveries stem from Mediterranean countries. By contrast, the vast majority of lapwings from Northwestern Europe and Fennoscandia spend the winter in France, on the Iberian peninsula or in Northern Africa [5]. Only 1 to 3% of lapwings from Northwestern Europe and Fennoscandia were recovered on the British Isles, but more than 50% of the recoveries from both continental populations were found in Western Mediterranean countries. British lapwings tend to migrate less towards continental Europe than previously documented [5, 40]. Shorter migration routes may be associated better body condition and improvement in life-history traits [39] may thus be an explanation for the high survival probabilities of British lapwings. Differences in survival between Northwestern and Fennoscandian lapwings are more difficult to explain. Migration routes are longer for Fennoscandian lapwings, but it is difficult to imagine that this mechanism alone results in lower survival in this long-lived species [41–43]. Differences could also be linked to factors operating on the breeding grounds such as predation or habitat quality [44].

### Temporal variation in recovery and reporting rates

Recovery probabilities declined strongly over time in the Western Mediterranean region, the primary hunting area. Such trends have also been observed in other species [29] and were presumed to reflect behavioral changes of people reporting ring recoveries. The observed decline in lapwing recovery probabilities is likely related to the closure of the French ringing center during the 1990s. People reporting a ring usually receive a life history sheet of the bird as an acknowledgement for their participation which motivates them to continue reporting. However, no information was sent back to hunters during the ten-year closure which resulted in an ongoing reduced willingness to report recoveries, in particular in France (B. Trolliet, *pers comm*.). Even after the French ringing center started to work properly again (from 2002 onwards) and processed the information waiting from the 1990s, less than 50 recoveries were recorded each year. If the numbers of recoveries remain at this level, future inference about survival from ring-recoveries might be compromised. We suggest that simple adaptation based on experiences from North America such as providing a toll-free phone number or a ring-reporting website (www.ring.ac), or more active communication with the hunter community may help to improve the reporting behavior [29].

Information about the propensity of hunters to report rings and the percentage of shot but not retrieved individuals is weak or simply missing in Europe [2]. We had to assume that lapwings were reported at similar rates as mourning doves from North America (reporting rate of 0.35 and retrieval rate of 0.75) which might be questionable and may have resulted in potential bias. We assessed the sensitivity of the kill rate to different values of reporting and retrieval rates (Table A in S8 File) and found that the kill rate was less than 0.1 even in the worst-case scenario (reporting rate = 0.1 and retrieval rate = 0.5), suggesting that our assumption was valid and our estimates were reasonable.

### Hunting mortality and kill rate

Using information about the cause of death stored in the EURING databank, we estimated the relative importance of hunting mortality for lapwings. The average proportion of death due to hunting was low and fairly constant over time for both age classes and for each ringing area, except in the last decade. Consistent with the migratory behavior of lapwings and the ban of lapwing hunting in the United Kingdom and in Ireland, the estimated proportion of hunting mortality of birds from the British Isles was close to 0.

The probability that a lapwing is killed by a hunter (kill rate) in a given year was less than 0.05 over the study period of 50 years. Adult mallards (*Anas platyrhynchos*) and adult greater snow geese (*Chen caerulescens atlantica*), North-American migrants with similar survival probabilities than lapwings (0.60–0.80 and 0.70–0.80 for mallard and snow goose, respectively [45, 46]), have similar kill rates (0.06–0.12 and 0.04–0.06 for the period 1960–2010 respectively [46, 47]) than AFY lapwings. Kill rates of FY individuals of these two species were higher (0.12–0.31 and 0.15–0.25, respectively) than that of lapwings [46, 47]. Despite these higher kill rates mallard populations remain stable and populations of greater snow goose were even able to increase [46, 47]. This suggests that healthy populations can support a higher level of harvesting than lapwings experienced.

The kill rate of lapwings declined slightly over time, which may have resulted from changes in regulations reducing hunting opportunities or due to a decreasing number of hunters (i.e. France) [48, 49]. Both age classes of lapwings experienced similar kill rates, indicating no differential selection by hunters. This contrasts with other species where younger individuals are often more vulnerable to hunting that older individuals [50, 51].

The kill rates were similar for the Fennoscandian and North-western European lapwing populations, suggesting that both populations were subjected to the same hunting pressure. The observed difference in survival between these two populations is thus likely related to other causes of mortality than hunting, such as differential predation on the breeding grounds.

## Conclusion

Lapwing populations increased after 1950 across most European countries (Appendix 1 in [5]), but experienced a marked decline since the 1970-80s [52]. Our study showed that survival probabilities of lapwings of both age classes and from three major European breeding regions remained fairly constant over time and even tended to increase slightly, while the estimated kill rates and the proportion of harvested individuals were low and tended to decline over time. These results suggest that hunting was not a primary mechanism for the decline of European lapwing populations since the 1980s. It has previously been proposed that major reasons for the decline of lapwing populations are changes in land use that resulted in declines of productivity [8, 53, 54]. However, our study does not support the claim that lapwing population dynamics is completely unaffected by hunting. To investigate finer-scaled impacts of hunting or to identify the demographic reason of the change in population trends we need to study whether hunting mortality is an additive or a compensatory source of mortality [55] and to perform retrospective population analyses based on population models that include all demographic processes.

## Supporting Information

S1 File List of ringing schemes that provided the ringing and recovery data. Includes a table summarizing origin of ringing data and a figure illustrating locations of recoveries by area of ringing.
(PDF)

S2 File Implementation of the multievent cause-specific mortality model in E-SURGE. We provided the full procedure to implement this model in program E-SURGE.
(PDF)

S3 File Multievent Seber model. Brief description of the model used to estimate survival and recovery probabilities while taking into account uncertainty in the recovery process.
(PDF)

S4 File Review of reporting rates and crippling loss rates of Mourning doves. Tables summarizing reporting rates and crippling loss rates of Mourning doves from the literature.
(PDF)

S5 File Point estimates (± SE) of several demographic parameters for lapwing populations from 1960 to 2010. (PDF)

S6 File On the use of recoveries with unknown source of death in the standard cause-specific mortality model. Comparison of mean and standard errors survival and proportion of death due to hunting between the multievent cause-specific mortality model and the standard cause-specific mortality model [16] where recoveries with unknown cause of death where either assigned to another cause of death (i.e. not harvested) or discarded.
(PDF)

S7 File Comparison of parameter estimates obtained from the multievent cause-specific mortality model and a multievent Seber model. (PDF)

S8 File Comparison of kill rates computed from different values of reporting and retrieval rates. (PDF)

S9 File Dataset. Encounter histories of ringed lapwings formatted for the multievent cause-specific mortality model adopted in our study. Lapwings were ringed from 1960 to 2010 in Denmark, Finland, Germany, Great-Britain, Netherlands, Norway and Sweden.
(TXT)
