# Supplementary material for: Investigating Rates of Hunting and Survival in Declining European Lapwing Populations
Source: PLoS One. 2016 Sep 29;11(9):e0163850. doi: 10.1371/journal.pone.0163850 (PMC5042549; doi:10.1371/journal.pone.0163850)
Supplement: S1 File — Includes a table summarizing origin of ringing data and a figure illustrating locations of recoveries by area of ringing. (PDF) [file pone.0163850.s001.pdf]

S1 List of ringing schemes that provided the ringing and recovery data.

Table A: Summary of ringing schemes that provided ringing data

Country	Scheme	Head
Denmark	Copenhagen Bird Ringing Centre	Kasper Thorup
Finland	Finnish Museum of Natural History	Jari Valkama
Germany	1- Institut für Vogelforschung "Vogelwarte Helgoland"	Franz Bairlein
	2- Beringungszentrale Hiddensee Hiddensee Bird Ringing Centre State Office for Environment, Nature Conservation and Geology (LUNG)	Ullrich Köppen
Great-Britain	British Trust for Ornithology	Jacquie Clark
Netherlands	Vogeltrekstation	Henk van der Jeugd
Norway	Bird Ringing Centre Museum Stavanger	Alf Tore Mjös
Sweden	Bird Ringing Centre Swedish Museum of Natural History	Thord Fransson

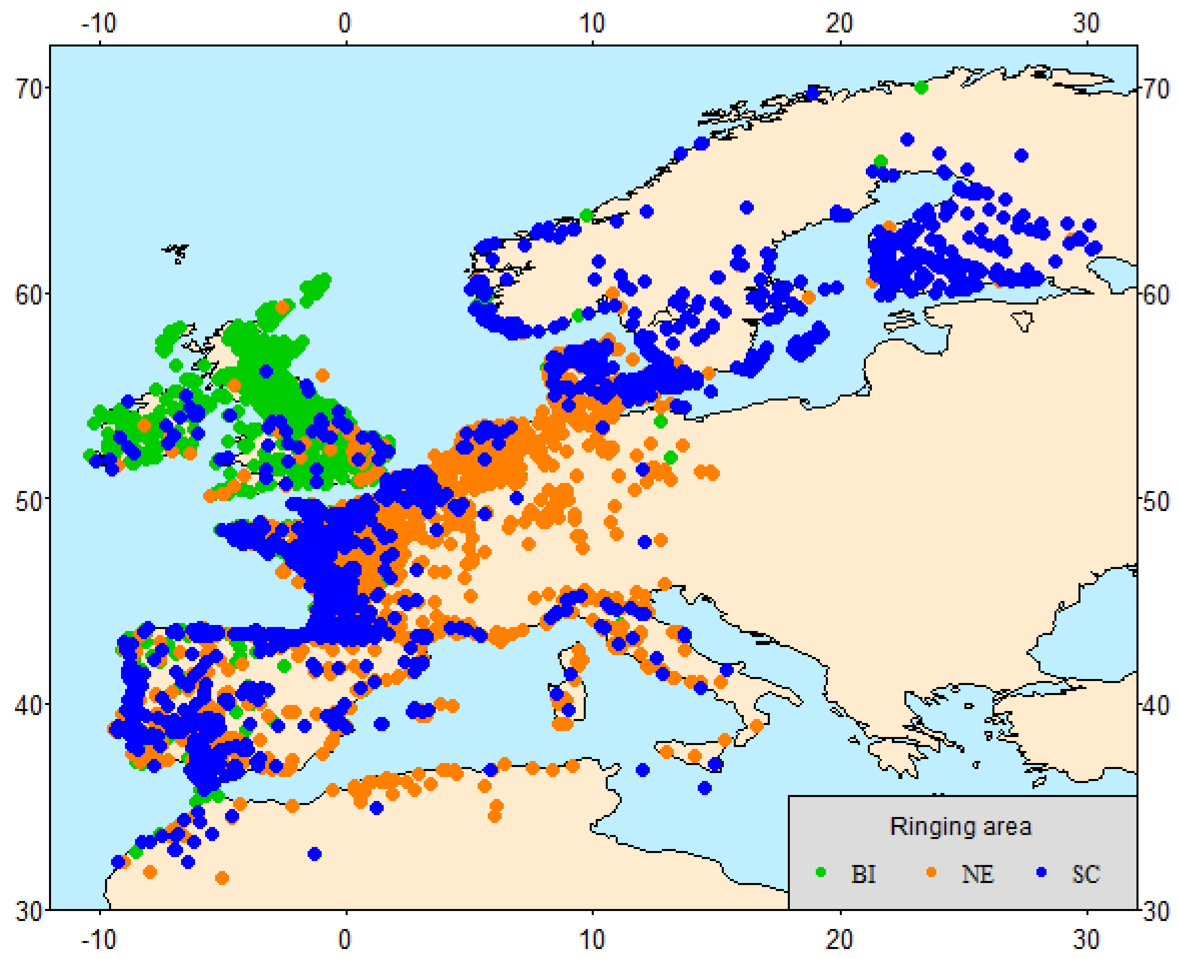

Figure A: Map of lapwing recovery data from the seven ringing countries. Lapwings were ringed as chicks from 1960 to 2009 and recovered from 1960 to 2010.

BI = British Isles, NE = North-western Europe, SC = Fennoscandia
