# Supplementary material for: Investigating Rates of Hunting and Survival in Declining European Lapwing Populations
Source: PLoS One. 2016 Sep 29;11(9):e0163850. doi: 10.1371/journal.pone.0163850 (PMC5042549; doi:10.1371/journal.pone.0163850)
Supplement: S2 File — We provided the full procedure to implement this model in program E-SURGE. (PDF) [file pone.0163850.s002.pdf]

S2 Implementation of the multi-event model in E-SURGE.

The multi-event framework relates the recovery events to the hidden biological states. The possible states of an individual are “A” alive, “NDh” newly dead due to hunting, “NDo” newly dead due to other causes and “D” dead. Our model estimates two kinds of parameters: transition probabilities between states and encounter probabilities linking events to biological states. The transition is decomposed into two steps, following Schaub and Pradel (2004), with survival and then proportion of mortality for each source of death. Here is the matrix corresponding to the survival step:

$$\begin{array}{c} \\ A \\ NDh \\ NDo \\ D \end{array} \begin{array}{c} \begin{array}{ccc} \boldsymbol{A} & ND & \boldsymbol{D} \end{array} \\ \begin{bmatrix} S & 1-S & 0 \\ 0 & 0 & 1 \\ 0 & 0 & 1 \\ 0 & 0 & 1 \end{bmatrix} \end{array},$$

where rows and columns of the matrix represent pre- and post-transition states, respectively. An individual will survive with probability S and stay in the state Alive (A), or will die and enter the intermediate state Newly Dead (ND). The second step corresponds to the relative proportion of the difference sources of mortality:

$$\begin{array}{c} \\ \boldsymbol{A} \\ ND \\ \boldsymbol{D} \end{array} \begin{array}{c} \begin{array}{cccc} \boldsymbol{A} & \boldsymbol{NDh} & \boldsymbol{NDo} & \boldsymbol{D} \end{array} \\ \begin{bmatrix} 1 & 0 & 0 & 0 \\ 0 & \alpha & 1-\alpha & 0 \\ 0 & 0 & 0 & 1 \end{bmatrix} \end{array},$$

where α is the proportion of newly dead individual that died due to hunting.

The event probabilities correspond to the probabilities to find and report a ring of a dead lapwing depending on the circumstance of the death and the area of recovery, following the fate diagram (Fig).

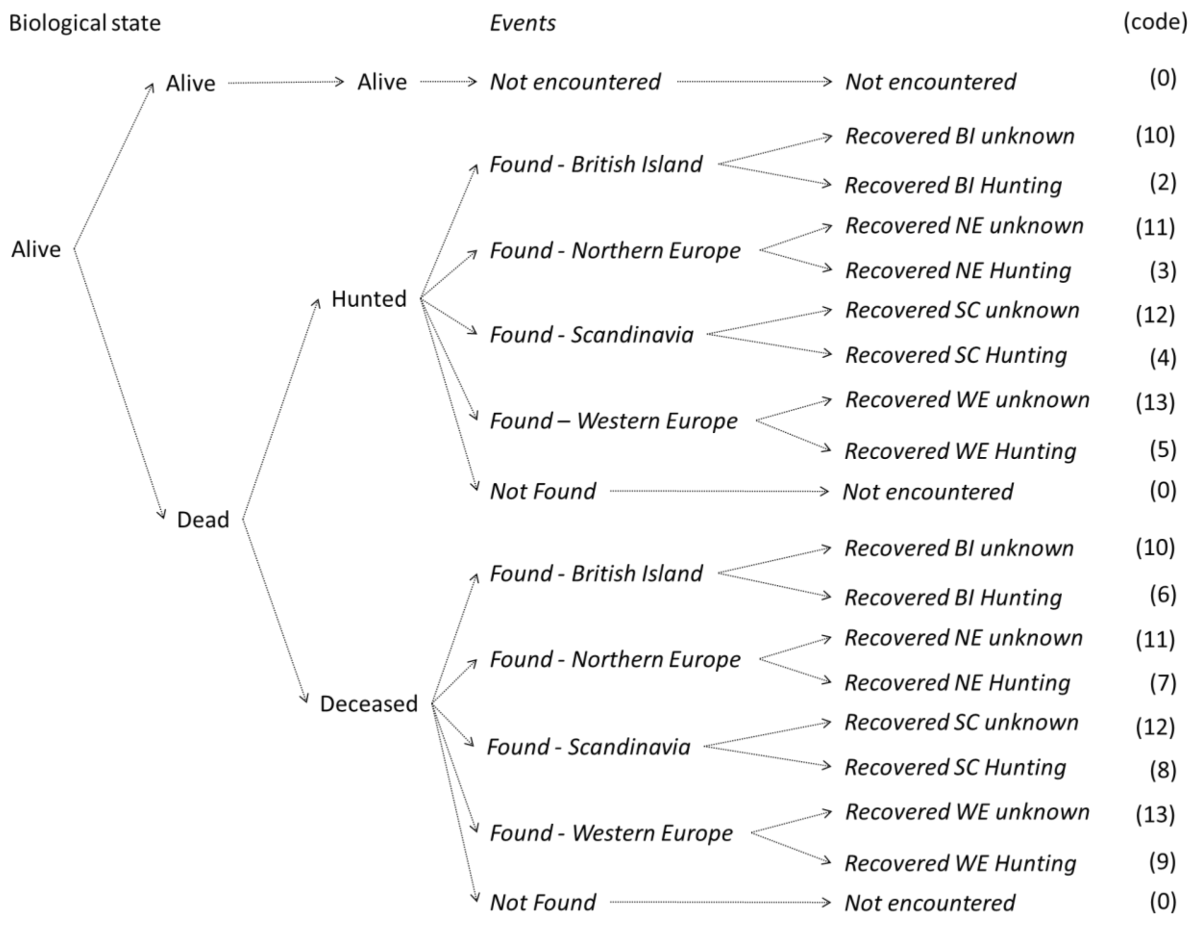

Figure A: Diagram of the fate of a ringed lapwing until it is recovered. The left part of the diagram represents the transition between biological states (survival then sources of death) of a ringed lapwing while the part written in italics represents what happens on the field (recovery with respect to location and then assignment of the cause of death). Numbers inside brackets represent the code used in the dataset for each event.

Due to the high number of recoveries with unknown cause of death, we decomposed the event probabilities into two steps: the recovery and the circumstance assignment steps. The first step links the biological states to the intermediate recovery event: the intermediate codes “2” to “5” correspond to individuals recovered after being dead due to hunting for each area and the codes “6” to “9” to individuals recovered after being dead due to other causes for each area.

$$
\begin{array}{c} \\ A \\ NDh \\ NDo \\ D \end{array}
\begin{array}{cccccccccc}
0 & 1 & "2" & "3" & "4" & "5" & "6" & "7" & "8" & "9" \\
\end{array}
$$

$$
\begin{array}{c} A \\ NDh \\ NDo \\ D \end{array}
\begin{bmatrix}
1 & 0 & 0 & 0 & 0 & 0 & 0 & 0 & 0 & 0 \\
1-\sum r_h^i & 0 & r_h^{BI}\delta_h & r_h^{NE}\delta_h & r_h^{SC}\delta_h & r_h^{WM}\delta_h & 0 & 0 & 0 & 0 \\
1-\sum r_o^i & 0 & 0 & 0 & 0 & 0 & r_o^{BI}\delta_o & r_o^{NE}\delta_o & r_o^{SC}\delta_o & r_o^{WM}\delta_o \\
1 & 0 & 0 & 0 & 0 & 0 & 0 & 0 & 0 & 0
\end{bmatrix}_t ,
$$

where rows correspond to the biological states, and columns to intermediate events. Alive individual can be captured only once, when they are marked for the first time at chick stage. Thus, the capture probability *p* is fixed to 1 for the first encounter and to 0 for subsequent occasions. A newly dead individual will be recovered with probability r_j^i depending on the area of recovery *j* and the cause of death *i*. In a second step, we estimated the probability δ^i that a recovery was assigned to the cause of death *i* determining the final code associated to the whole event (recovery + cause assignment, see also Fig. S2.1).

$$
\begin{array}{c} \\ 0 \\ 1 \\ "2" \\ "3" \\ "4" \\ "5" \\ "6" \\ "7" \\ "8" \\ "9" \end{array}
\begin{array}{cccccccccccccc}
0 & 1 & 2 & 3 & 4 & 5 & 6 & 7 & 8 & 9 & 10 & 11 & 12 & 13 \\
1 & 0 & 0 & 0 & 0 & 0 & 0 & 0 & 0 & 0 & 0 & 0 & 0 & 0 \\
0 & 1 & 0 & 0 & 0 & 0 & 0 & 0 & 0 & 0 & 0 & 0 & 0 & 0 \\
0 & 0 & \delta_h & 0 & 0 & 0 & 0 & 0 & 0 & 0 & 1-\delta_h & 0 & 0 & 0 \\
0 & 0 & 0 & \delta_h & 0 & 0 & 0 & 0 & 0 & 0 & 0 & 1-\delta_h & 0 & 0 \\
0 & 0 & 0 & 0 & \delta_h & 0 & 0 & 0 & 0 & 0 & 0 & 0 & 1-\delta_h & 0 \\
0 & 0 & 0 & 0 & 0 & \delta_h & 0 & 0 & 0 & 0 & 0 & 0 & 0 & 1-\delta_h \\
0 & 0 & 0 & 0 & 0 & 0 & \delta_o & 0 & 0 & 0 & 1-\delta_o & 0 & 0 & 0 \\
0 & 0 & 0 & 0 & 0 & 0 & 0 & \delta_o & 0 & 0 & 0 & 1-\delta_o & 0 & 0 \\
0 & 0 & 0 & 0 & 0 & 0 & 0 & 0 & \delta_o & 0 & 0 & 0 & 1-\delta_o & 0 \\
0 & 0 & 0 & 0 & 0 & 0 & 0 & 0 & 0 & \delta_o & 0 & 0 & 0 & 1-\delta_o
\end{array}
$$

For example, if a ringed bird is shot in France, its ring will be recovered with probability r_{WE}^h and recorded “dead due to hunting” with probability δ_h or recorded “unknown cause of death” with probability (1- δ_h).

1. Getting E-SURGE

Install E-SURGE following the indication from the Biostatistic group of the CEFE-CNRS team in Montpellier: http://www.cefe.cnrs.fr/fr/ressources/logiciels/34-french/recherche/bc/bbp/264-logiciels

A manual can also be downloaded from the same website:

http://www.cefe.cnrs.fr/images/stories/DPTEEvolution/biostatistiques/LOGICIELS/E-SURGE-MANUAL.pdf

The manual provides useful insights and advice for formatting the data and running a model. The format "HEADED" is recommended because it allows to name each column, which is especially useful for group or covariate columns. For instance, for each individual, we have a group effect for the ringing area, the column "$COV:Country".

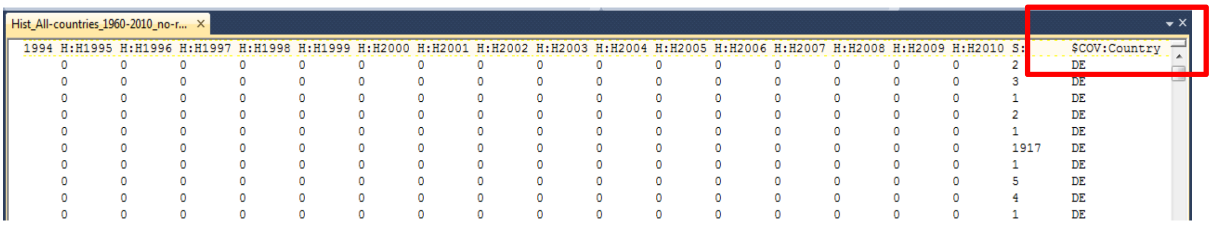

1994	H:H1995	H:H1996	H:H1997	H:H1998	H:H1999	H:H2000	H:H2001	H:H2002	H:H2003	H:H2004	H:H2005	H:H2006	H:H2007	H:H2008	H:H2009	H:H2010	S:	$COV:Country
	0	0	0	0	0	0	0	0	0	0	0	0	0	0	0	0	2	DE
	0	0	0	0	0	0	0	0	0	0	0	0	0	0	0	0	3	DE
	0	0	0	0	0	0	0	0	0	0	0	0	0	0	0	0	1	DE
	0	0	0	0	0	0	0	0	0	0	0	0	0	0	0	0	2	DE
	0	0	0	0	0	0	0	0	0	0	0	0	0	0	0	0	1	DE
	0	0	0	0	0	0	0	0	0	0	0	0	0	0	0	0	1917	DE
	0	0	0	0	0	0	0	0	0	0	0	0	0	0	0	0	1	DE
	0	0	0	0	0	0	0	0	0	0	0	0	0	0	0	0	5	DE
	0	0	0	0	0	0	0	0	0	0	0	0	0	0	0	0	4	DE
	0	0	0	0	0	0	0	0	0	0	0	0	0	0	0	0	1	DE

This format is useful because column names can be used when setting the model (see below).

2. Starting a session

Once the program is installed and the data formatted in one of the proper format (either Biomeco, MARK or Headed format), click on the "start" menu in the upper left of the E-SURGE windows.

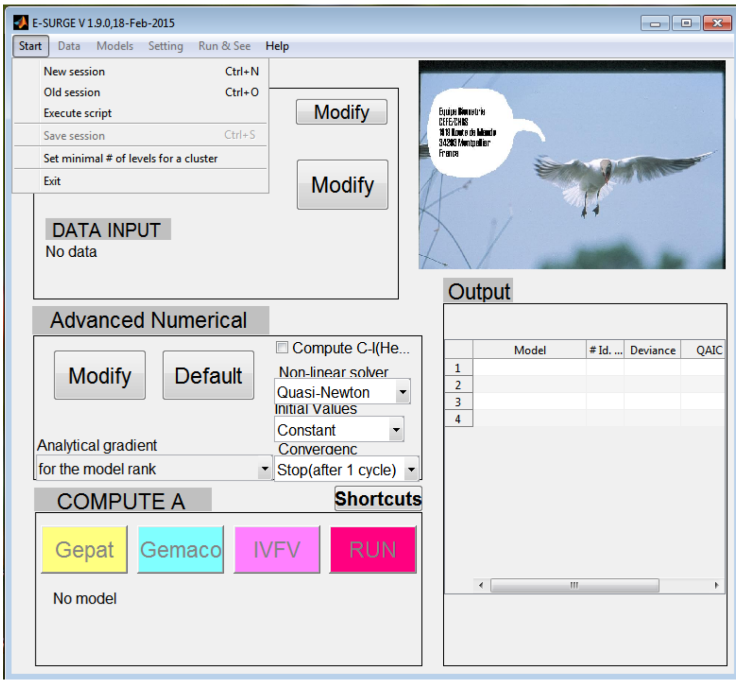

Then, click on "new sessions" to create the working session. Choose the folder where the session file and all model outputs will be saved.

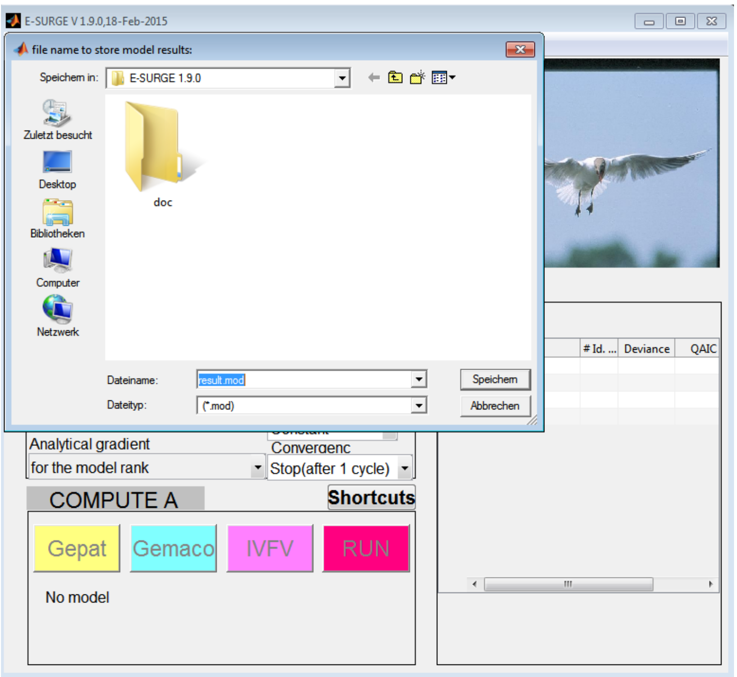

Load the data into the program: click on the "data" menu and choose the proper format.

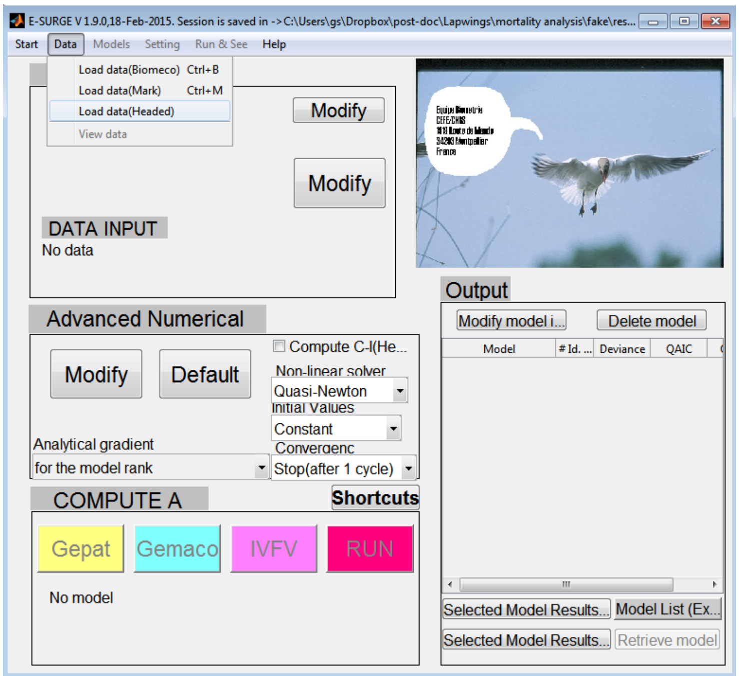

A pop-up window will open allowing the selection of the data file. As we use the Headed format, a pop-up window opens:

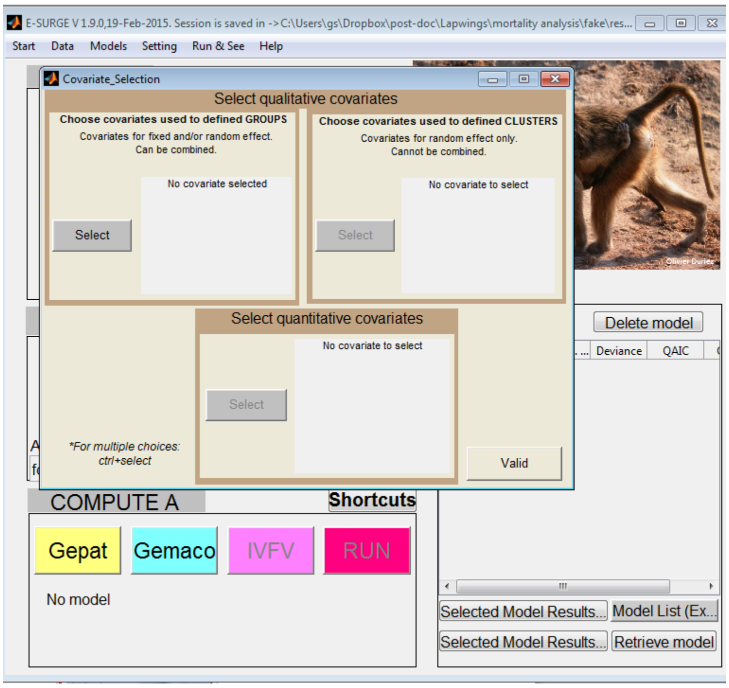

Now we can specify which column contains qualitative covariates: in our case, the country of ringing of individuals. Only the Upper-left "Select" is not grayed because we only have one column that defines a group.

Click on “Select”. A smaller pop-up opens where the Group Covariate can be selected. Here, we select the “Country”. Then, click on “Valid” in the lower-right part of the window.

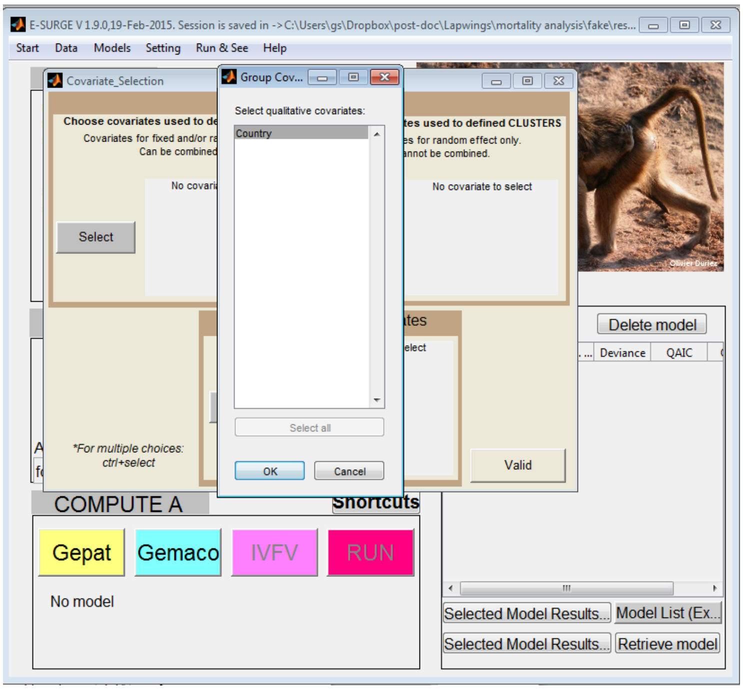

Once the data are loaded, you can check at their characteristic in the “Data” section of the E-SURGE window.

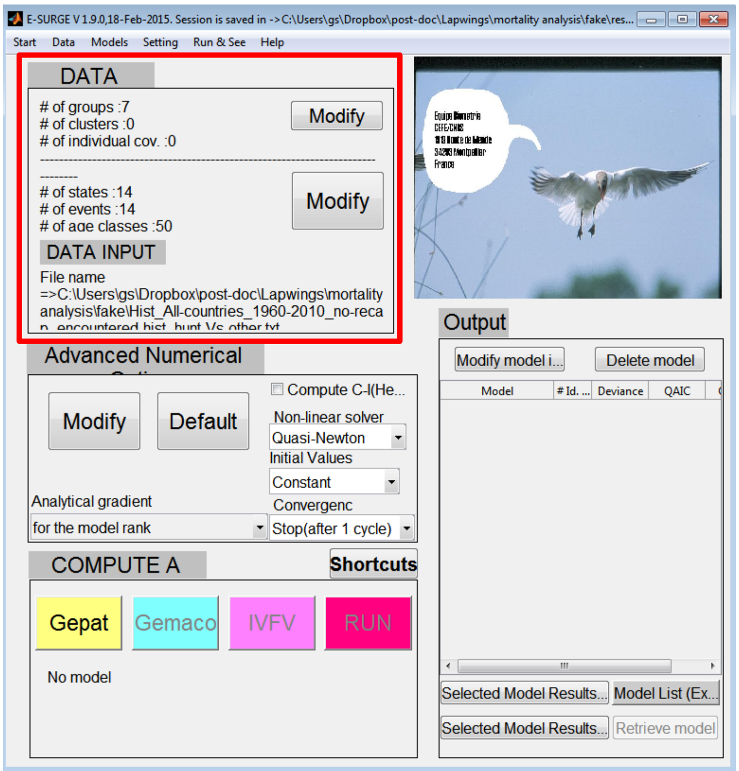

By clicking on “Modify”, you will be able to set the right number of age-classes, states and events for your model.

In the present study, the number of age-classes is 2 (First-year or After first-year), we have 4 states (A, NDh, NDo, D) and 14 events.

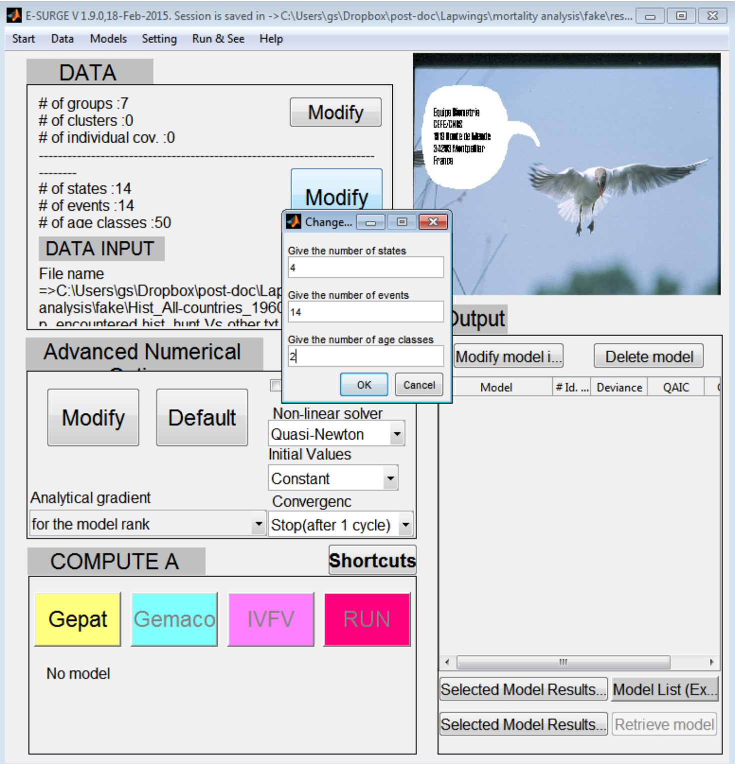

You may notice that only the yellow button is not grayed at this stage.

3. GEPAT

The next step is to enter the matrices of the model into E-SURGE, that is to define the basic structure of the model. To do so, click on the “GEPAT” yellow button (GEnerator of PATtern).

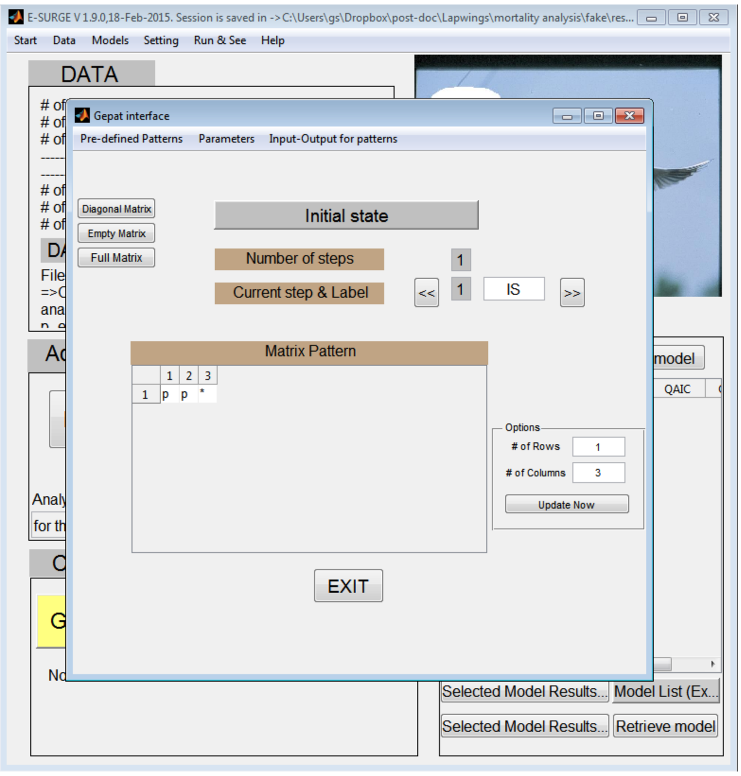

A new window opens allowing the definition of all matrices (initial state, transitions, observation). As a reminder, all matrices are row-stochastic in E-SURGE, thus all the components of a given row sum to 1. If only one parameter is present in a row, it will be fix to 1. If two parameters are present, a will be estimated (e.g “p”), and the other one will be his complement “1-p”. The complement symbol is “*”, while parameter symbols are letters of your choice. A fixed zero (0) in the matrix is coded by “-“.

The first matrix to set up is the “Initial State” matrix. This is a matrix to define in which states the individuals are when they are marked. The program assumes that the last state is the dead state, thus, the matrix has only 1 row and 3 columns:

$$\begin{array}{ccc} A & NDh & NDo \\ 1 & 0 & 0 \end{array}$$

In the GEPAT format, this gives:

$$\begin{matrix} A & NDh & NDo \\ * & - & - \end{matrix}$$

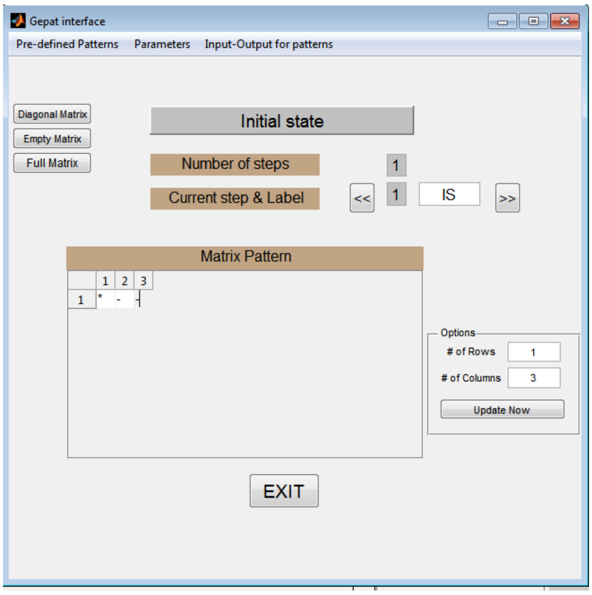

Once the Initial state matrix is specified, click on the grey button “Initial state”. Now a new window opens which allows the definition of the transition matrix.

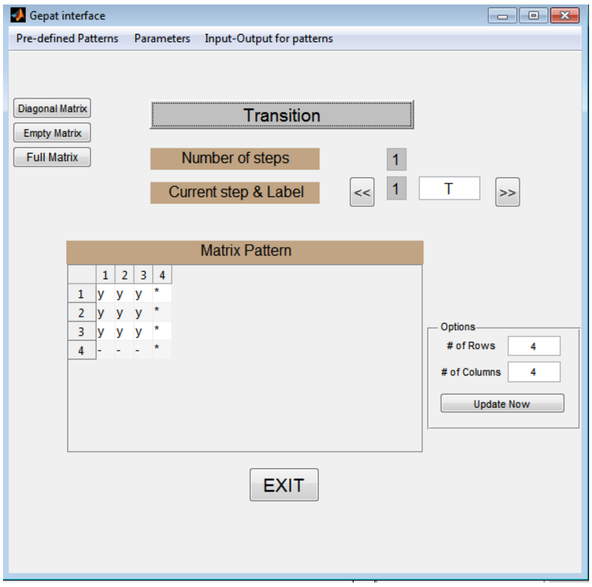

Here, we decompose the transitions among states in 2 steps: survival and then cause-specific mortality. Thus, we have to enter the number “2” for the number of step.

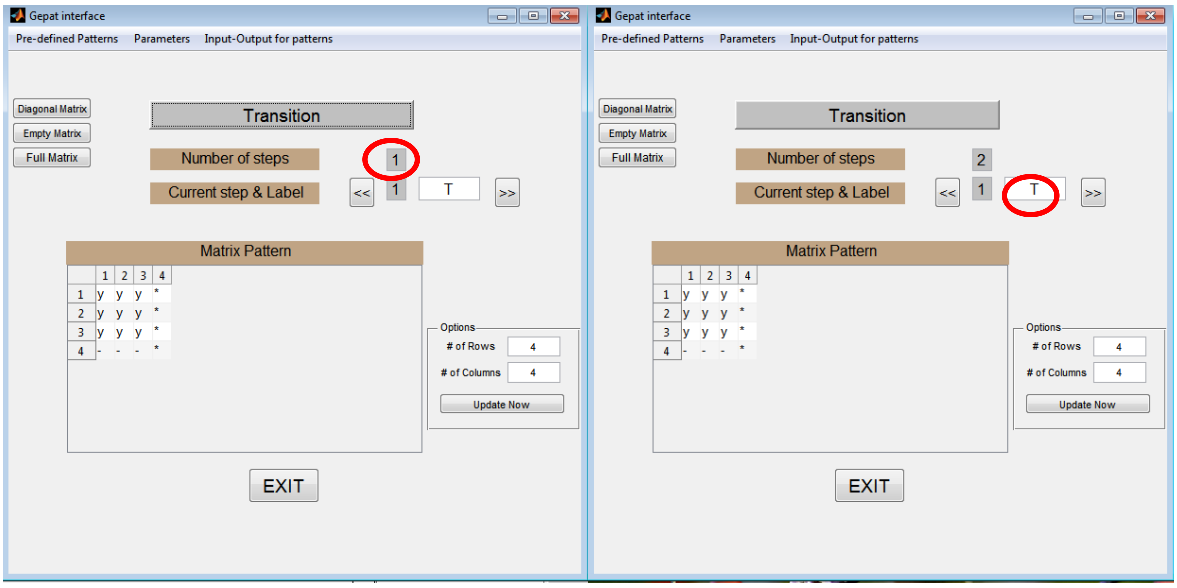

You can then give a specific name for each step: we use “surv” for step 1 (survival step) and “death” for step 2 (cause-specific mortality).

The survival matrix in GEPAT format is:

$$\begin{array}{c} \\ A \\ NDh \\ NDo \\ D \end{array}\begin{array}{c} \begin{array}{ccc} \boldsymbol{A} & ND & \boldsymbol{D} \end{array} \\ \begin{bmatrix} \varphi & 1-\varphi & 0 \\ 0 & 0 & 1 \\ 0 & 0 & 1 \\ 0 & 0 & 1 \end{bmatrix} \end{array} => \begin{array}{ccc} \varphi & * & - \\ - & - & * \\ - & - & * \\ - & - & * \end{array}$$

The survival matrix dimensions are 4 rows and 3 columns. We will use the “options” menu to update the number of rows and columns, then click on “Update Now”.

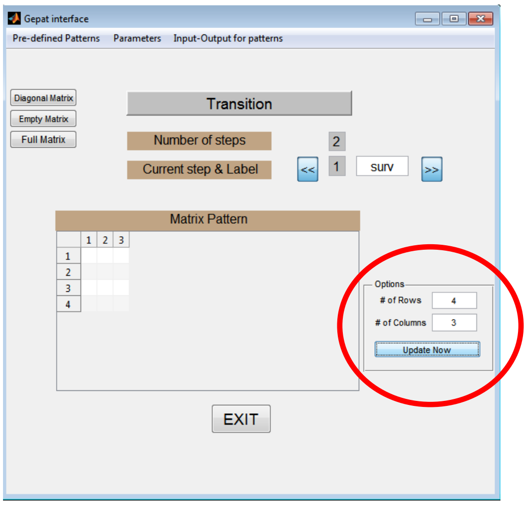

The matrix is empty and we have to fill it to match the survival matrix.

$$\begin{array}{ccc} phi & * & - \\ - & - & * \\ - & - & * \\ - & - & * \end{array}$$

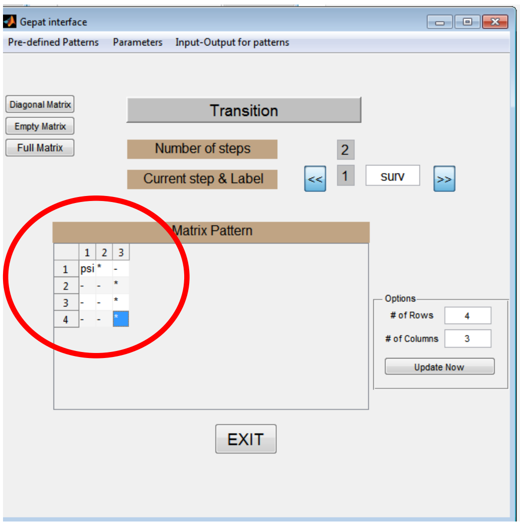

Then, click on the right arrow to open the second window the define the transition matrix of the second step. The new window is then empty, and the default name is “T”.

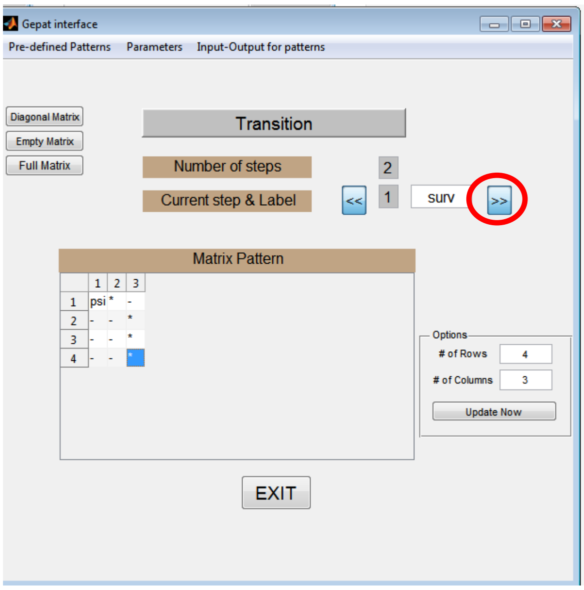

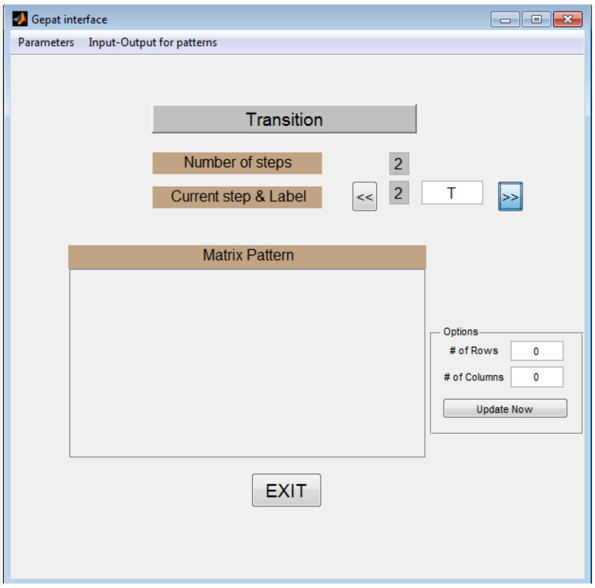

Following the same process, you can change the name from “T” to “death”. Then, update the matrix characteristics to match the source of death matrix (number of rows: 3, number of columns: 4). Click on “Update Now”.

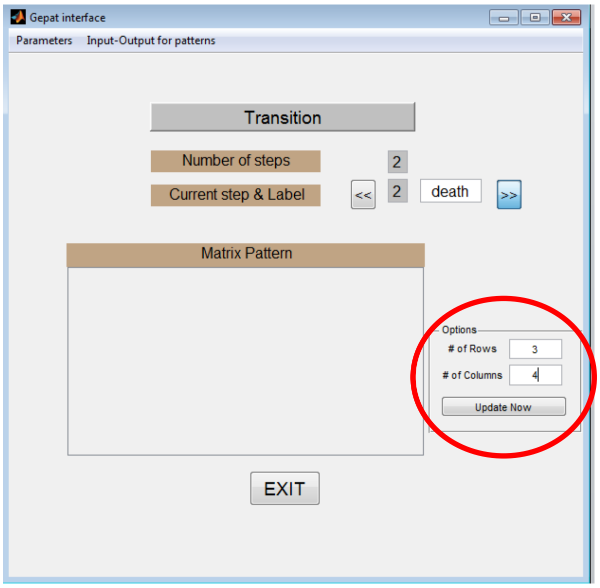

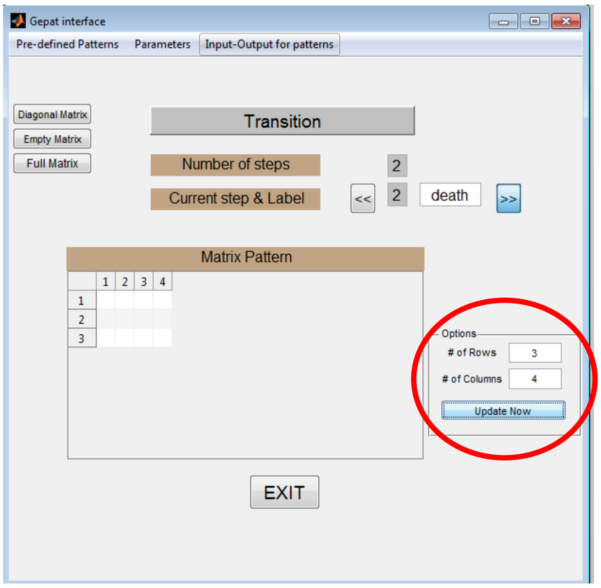

The matrix element is empty. Just fill it correctly based on the matrix:

$$\begin{array}{c} \\ \boldsymbol{A} \\ ND \\ \boldsymbol{D} \end{array} \begin{array}{c} \begin{array}{cccc} \boldsymbol{A} & \boldsymbol{NDh} & \boldsymbol{NDo} & \boldsymbol{D} \end{array} \\ \begin{bmatrix} 1 & 0 & 0 & 0 \\ 0 & \alpha & 1-\alpha & 0 \\ 0 & 0 & 0 & 1 \end{bmatrix} \end{array}$$

$$=> \begin{array}{cccc} * & - & - & - \\ - & a & * & - \\ - & - & - & * \end{array}$$

The process to enter transition matrices is now completed.

Click on the button “Transition” to access the last matrix that needs to be defined, the event (observation) matrix.

The event matrix is a matrix with 4 rows (one for each state) and 14 columns (one for each event).

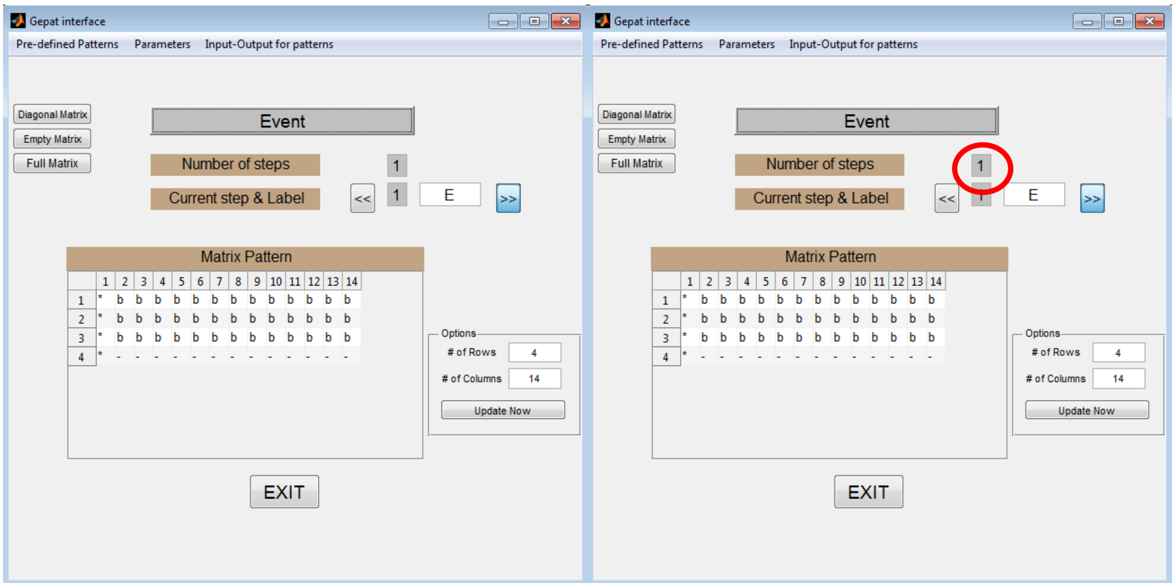

We will decompose the event matrix in 2 steps, the spatial recovery and the assignment.

First, enter the desired number of steps

We can rename the first step from the default “E” to “recov” to remind this is the recovery step.

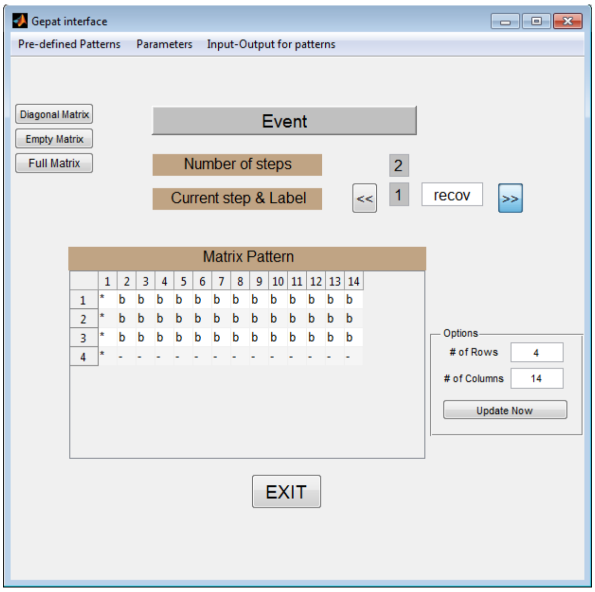

Enter the right matrix (4 rows but 10 columns, see beginning of appendix S2):

$$\begin{array}{cccccccccc} * & p & - & - & - & - & - & - & - & - \\ * & - & r & r & r & r & - & - & - & - \\ * & - & - & - & - & - & r & r & r & r \\ * & - & - & - & - & - & - & - & - & - \end{array}$$

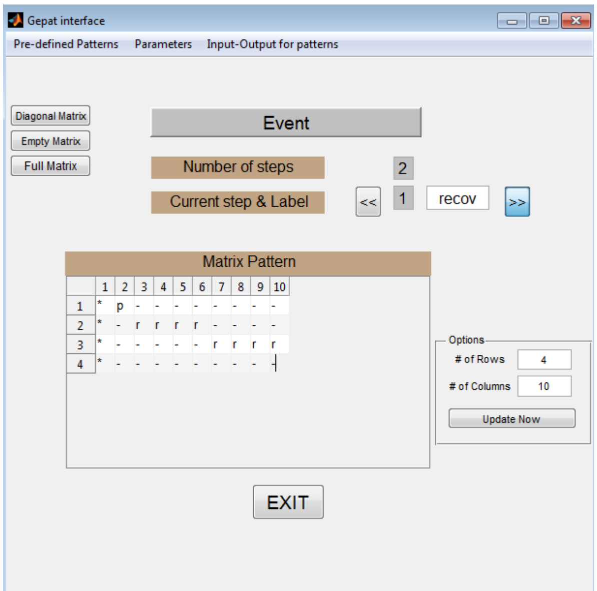

Then, click on the arrow to which allows the definition of the second step.

Again, you can change the name. Here, we will use “assign” to remind that this is the assignment step (whether a recovery was recorded under the correct cause of death or to the Unknown category).

Then update the matrix dimensions: 10 rows (to match the intermediate recovery events) and 14 columns (for each final event; see earlier in appendix S2 for the derivation of this matrix).

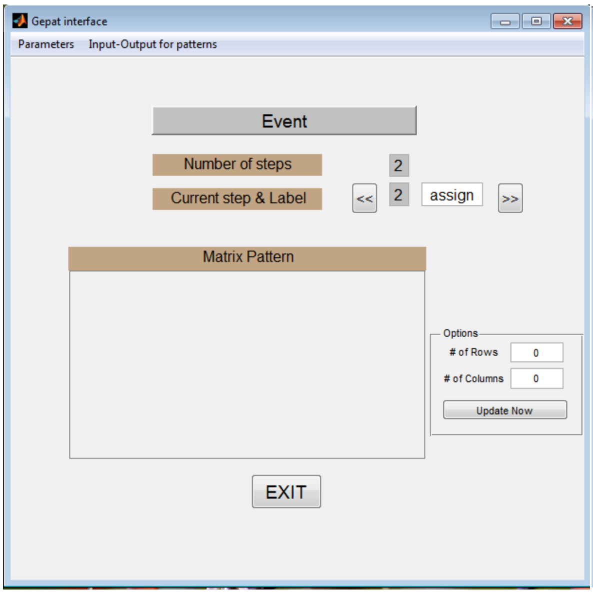

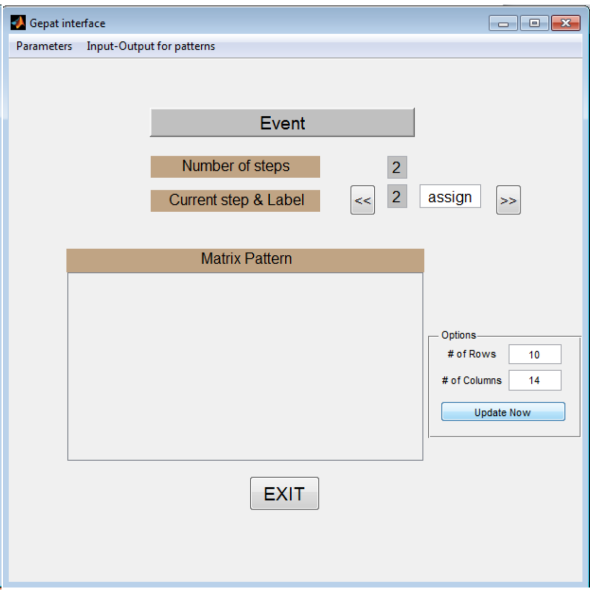

The second matrix refers to the assignment: (i) hunting recovery will be either coded as recovery due to hunting (final events 2 to 5 depending on the recovery area) or recovery due to unknown circumstance (10 to 13); (ii) recovery due to another cause will either correctly assign (events 6 to 9) or to unknown circumstances (10 to 13).

The corresponding matrix in GEPAT format is:

$$
\begin{matrix}
* & - & - & - & - & - & - & - & - & - & - & - & - & - \\
- & * & - & - & - & - & - & - & - & - & - & - & - & - \\
- & - & \delta & - & - & - & - & - & - & - & * & - & - & - \\
- & - & - & \delta & - & - & - & - & - & - & - & * & - & - \\
- & - & - & - & \delta & - & - & - & - & - & - & - & * & - \\
- & - & - & - & - & \delta & - & - & - & - & - & - & - & * \\
- & - & - & - & - & - & \delta & - & - & - & * & - & - & - \\
- & - & - & - & - & - & - & \delta & - & - & - & * & - & - \\
- & - & - & - & - & - & - & - & \delta & - & - & - & * & - \\
- & - & - & - & - & - & - & - & - & \delta & - & - & - & *
\end{matrix}
$$

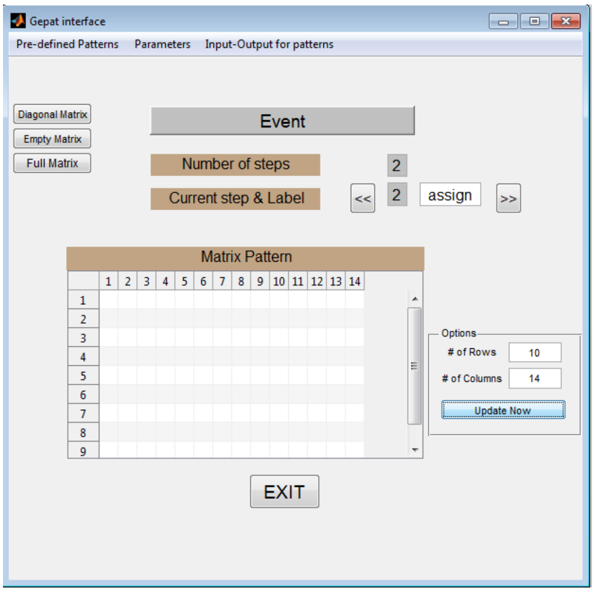

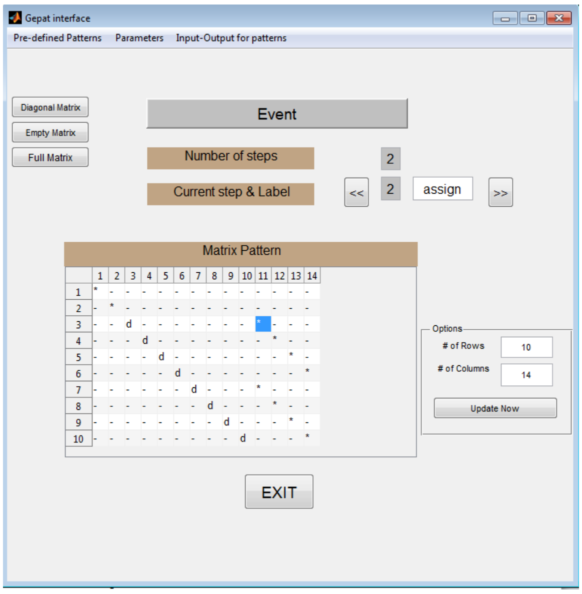

Once this is done, we (may) want to save this long work: 1- Click on “Input-Output for patterns” (this menu can be used also to load the saved pattern). 2- Click on “save file with patterns”. You can then give a name to a text file where the defined matrices are stored.

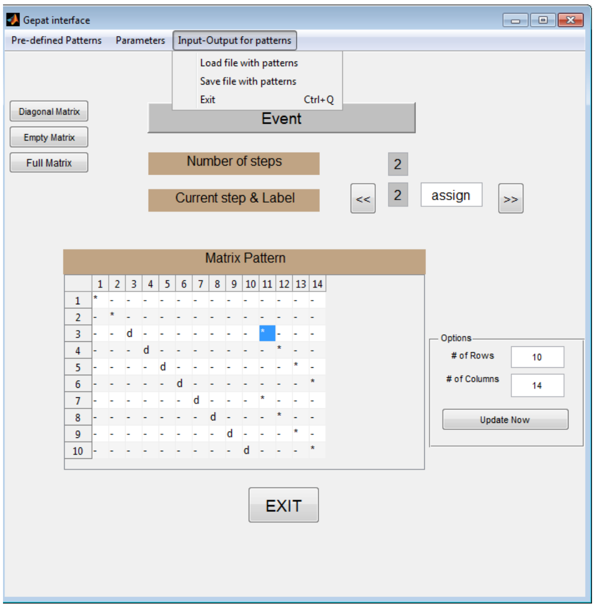

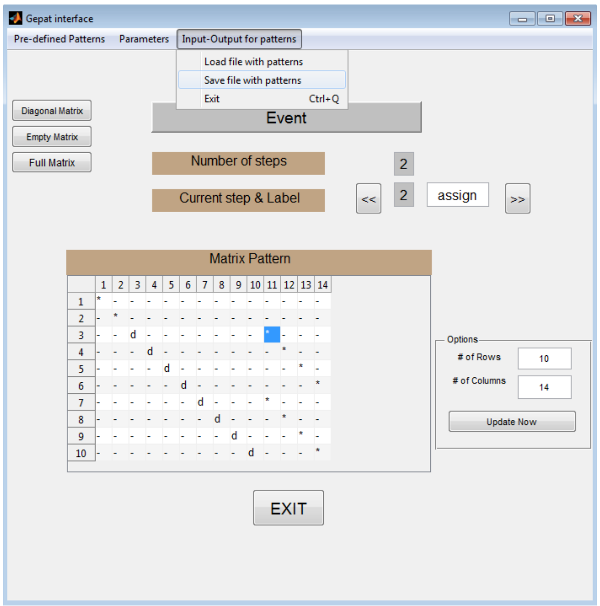

Now the GEPAT step is completed (the basic structure of the model is defined) and you click on the “EXIT“ button to return to the main window.

Now, E-SURGE knows the data and the matrices. A new button becomes avaiable, the blue one named “GEMACO”. This will allow setting constraints on parameters in the matrices, e.g. to specify whether survival depends on time, age, etc.

4. GEMACO

We will fit the initial model here: $S_{[Reg*a*y5]}, \alpha_{[Reg*a*y3]}, r_{[Hunt(law*y5),Other(y5)]}, \delta_{[cause]}$

Click on “GEMACO”. A new window opens.

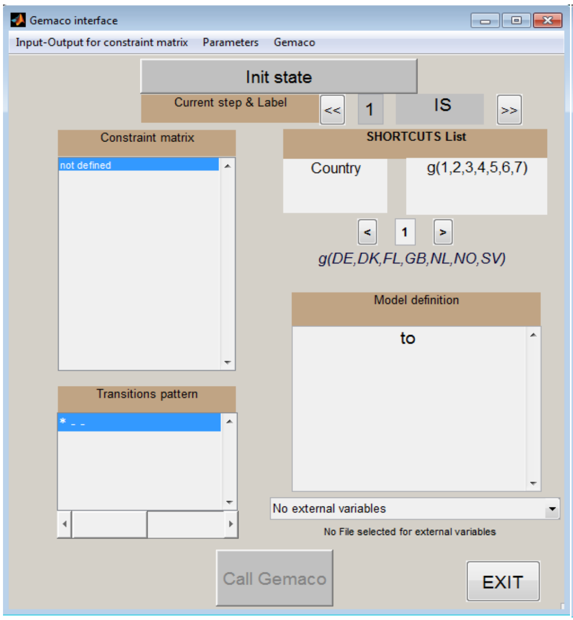

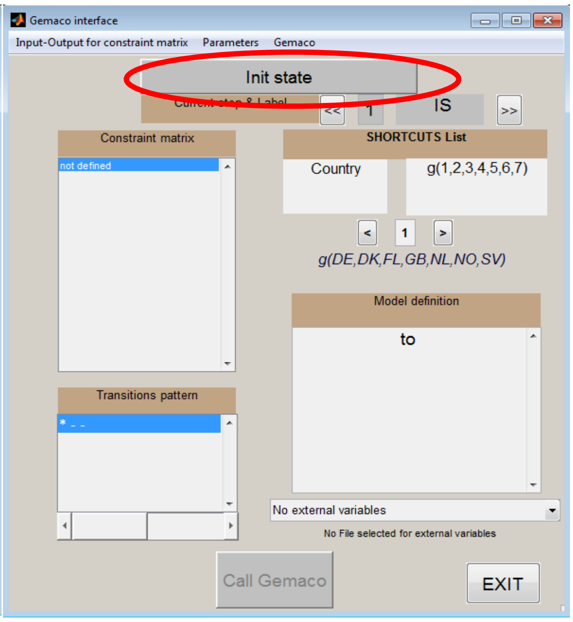

You are on the GEMACO interface. You can select on which kind of parameters you would like to set constraints by clicking on the great button in the upper part of the window (as in the GEPAT interface).

Because, we know with certainty the initial state of all individuals when they are marked, we have nothing to do with the Initial Step matrix. Thus, click on “Initial State” to get to the GEMACO interface for the transitions.

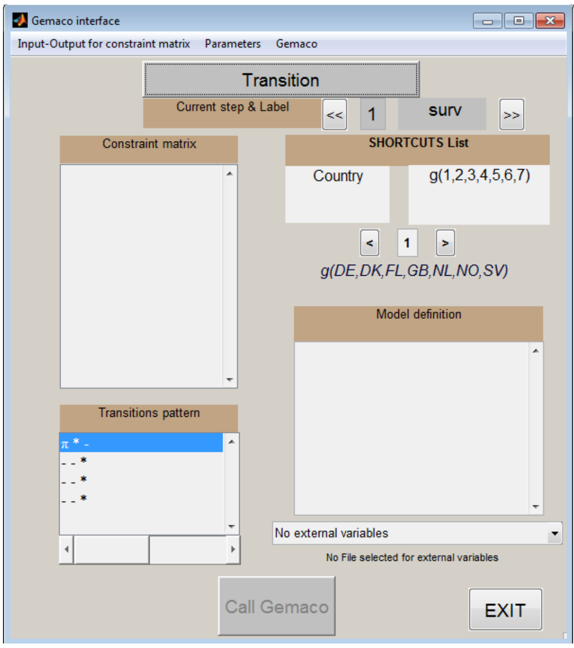

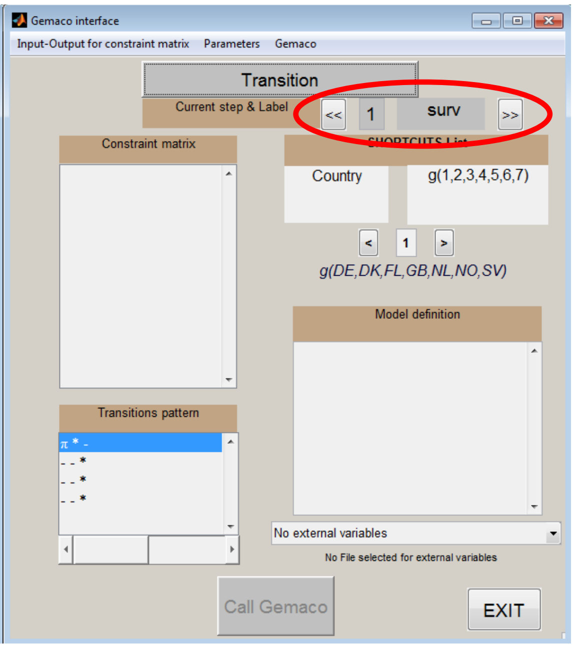

We are on the interface of step 1 of the transition stage, thus on the survival step. Using the arrow, you can switch between the different steps of the stage.

Remember that transitions are defined from occasion *t* to occasion *t+1*. Thus, we will refer to "interval" rather than to "occasion". Interval 1 is the interval between occasion 1 and occasion 2, and so on.

For survival, we want to set the following constraints:

- An age effect to specify different survival of the first year and of after the first year. This is simply doable by using the default shortcut "a" (see E-SURGE manual for a list of all default shortcuts). As we defined earlier than we only consider 2 age-classes, shortcut "a" will create automatically an age effect with 2 levels (A1, A2).
- A ringing area effect to produce different estimates for each ringing area: we will use the shortcut "Country" to create this effect. Country has 7 levels (one for each ringing country). We want to make several levels identical: Denmark with Germany and Netherlands, Finland with Sweden and Norway.

  The interface reminds you that the shortcut Country is g(1,2,3,4,5,6,7) in the "SHORCUT List" part. Each number refers to one level. Corresponding with the data is below this section "g(DE,DK,FL,GB,NL,NO,SV)". Thus, g(1) refers to the group "DE" = Germany, g(3) to "FL"= Finland, etc.

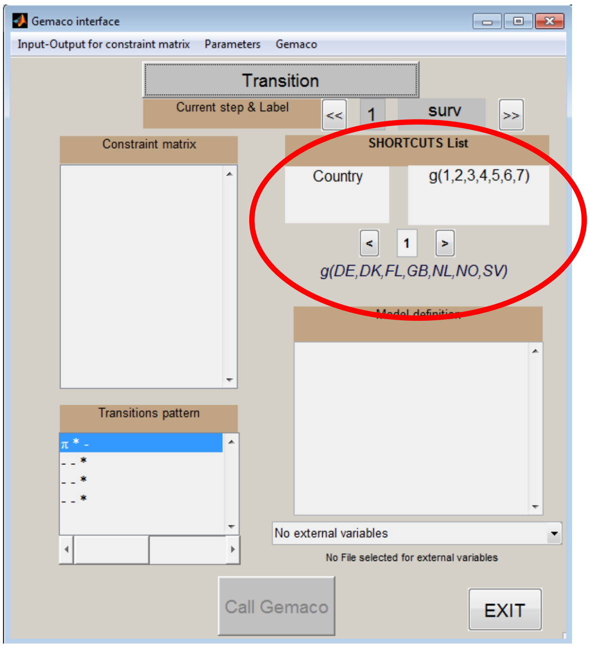

We want to pool several countries: g(DE DK NL,FL NO SV,GB).

In GEMACO language, this is translated into: Country(1 2 5,3 6 7,4).

- A time period effect (5 years period): the default shortcut "t" specifies a completely time-dependent model (different estimate for each interval). In our model, we want to set equality of survival within 5-years periods. Thus, survival during interval 1 shall be the same than during interval 2, 3 4 and 5. We can pool time intervals with command *t(1 2 3 4 5)*. To distinguish from the next 5 intervals, we just insert a comma "," between the lists:

  t(1 2 3 4 5,6 7 8 9 10, 11 12 13 14 15, 16 …, 46 47 48 49 50). To avoid such a long sentence, you can define shortcut (see manual). We created a shortcut named "an5" to such effect of 5-year period.

Finally, the complete sentence for survival is the interaction between all 3 constraints. An interaction is symbolized by ".". Therefore, we write the sentence in the section "Model definition" (copy/paste is allowed).

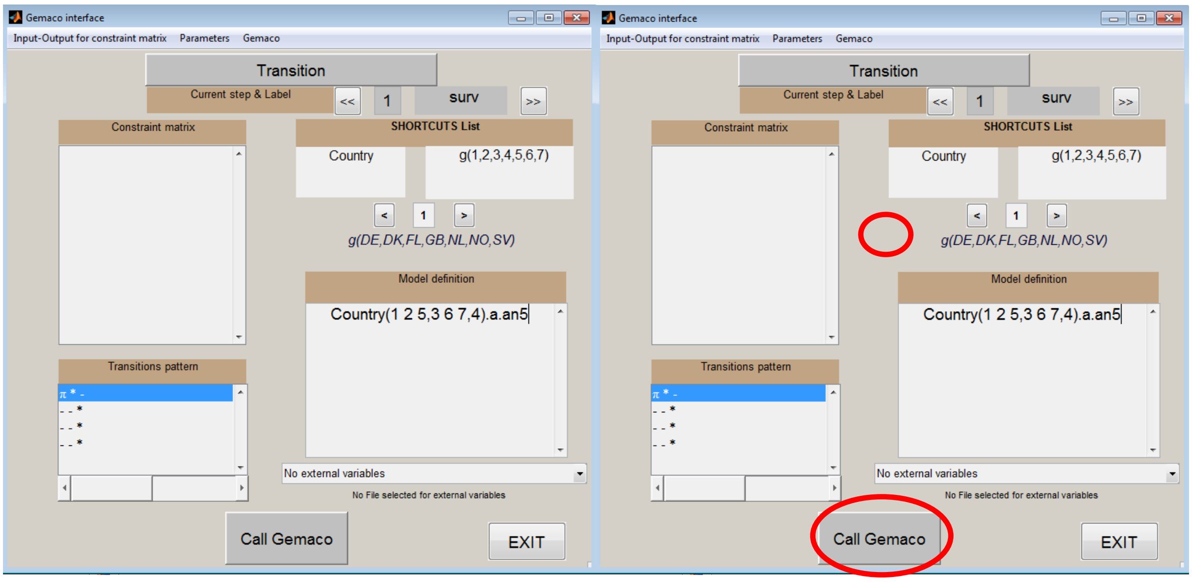

Now first click outside the Model definition part but still on the GEMACO interface, then on “Call Gemaco”. This will set the constraints on the survival matrix. Either the constraint matrix or a short message should appear in the “Constraint matrix” part of the GEMACO interface.

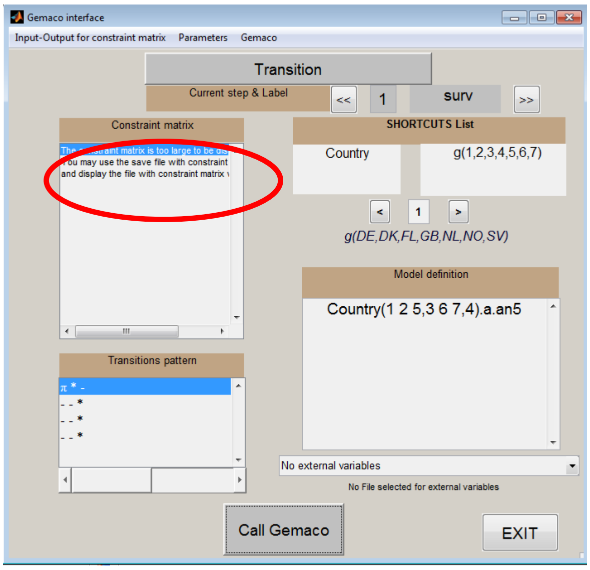

By the same process, we will constrain the second step of the transition and the event steps.

For the cause of death, the constraints are again an age effect, a ringing area effect and a time period effect, but now reduced to 3 years instead of 5. We create a shortcut “an3” for this particular constraint. The resulting sentence is:

Country(1 2 5,3 6 7,4).a.an3

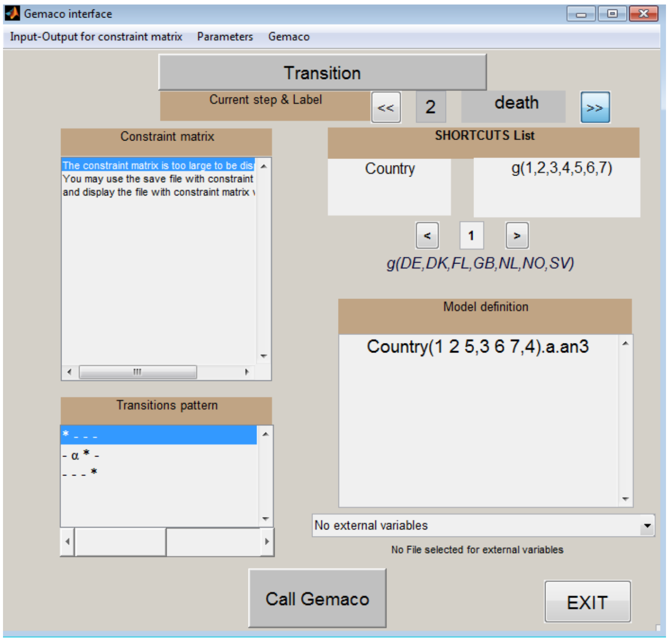

Then click on the big button to reach the Event interface.

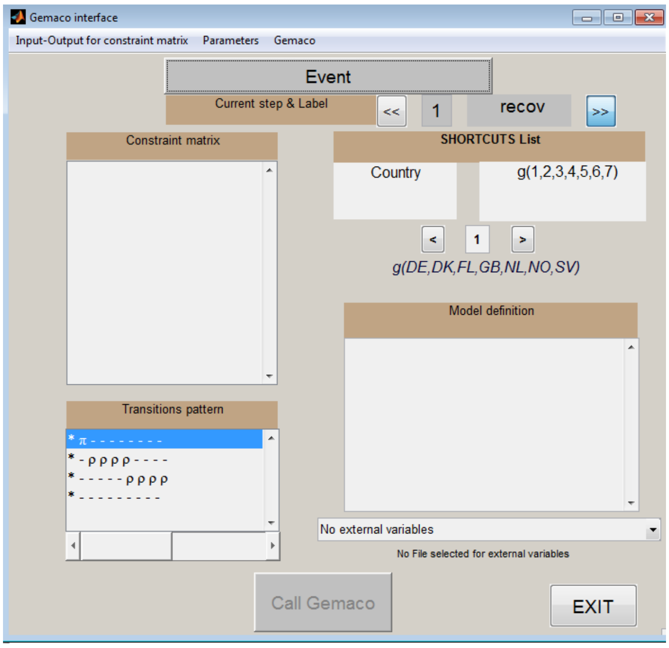

When dealing with events, we want to distinguish between the initial capture allowing the marking and all subsequent reencounters. E-SURGE knows 2 shortcuts to separate the first event of an individual (its marking) and the next ones.

"Firste" refers to the first event, and "nexte" refers to all the others. We use these shortcuts in an additive way with the operator "+". Note that "firste" refers to the first age class of the individuals (i.e. when birds are marked as chick), and "nexte" to the second age class (age1 onwards). Even if there is only one age-class in the model, "firste" will refer to age 1 (no age 0 in E-SURGE) and "nexte" defines an effect on age 2. This is a bit tricky, but keep it in mind.

The constraints on the recovery steps are a bit more complicated than those of the transitions. In the event matrix, several rows contain more than one parameters. Thus, we need other shortcuts (already defined in E-SURGE again) to refer to each unique parameter. The shortcut "f/from" allows to set constraint based on a particular row of the matrix while "to "refers to the column. In a matrix, you start FROM the row to arrive TO a specific column. For instance, the parameter on raw 1 (the live capture probability) is identified by *f(1)*. The

parameter in raw 2 and column 4 (i.e. the recovery probability due to hunting in the NE area) is identified by *f(2).to(4)*.

In our model, we will set different constraints to each kind of parameters:

- The recapture probability is constant over time and fixed to 0. This is specified in GEMACO with sentence *f(1)*;
- The recovery probabilities is different depending on time period (a created shortcut *ev5*)
    - Recovery due to hunting (row 2) in area WM is different to the other recovery areas (but equality among these areas):

      r(BI)=r(NE)=r(SC)#r(WM)

      thus "f(2).to(3)"= "f(2).to(4)"= "f(2).to(5)"# "f(2).to(6)".

      Therefore, you have to enter: f(2).to(3 4 5,6)
    - Recovery probabilities due to another cause (row 3) are identical in the different recovery areas, thus, no need to distinguish area-specific parameters

      r(BI)=r(NE)=r(SC)#r(WM) => f(3).to(7)= f(3).to(8)= f(3).to(9)=f(3).to(10)

      This is similar to f(3).to(7 8 9 10) or simply f(3)

Pooling all together, this gives:

firste+nexte.[f(1)+f(2).to(3 4 5,6).ev5+f(3).ev5]

The bracket allows factorization, thus everything inside brackets after "nexte" are related to "nexte". Again, click outside the part where you write the sentence and click on "Call Gemaco"

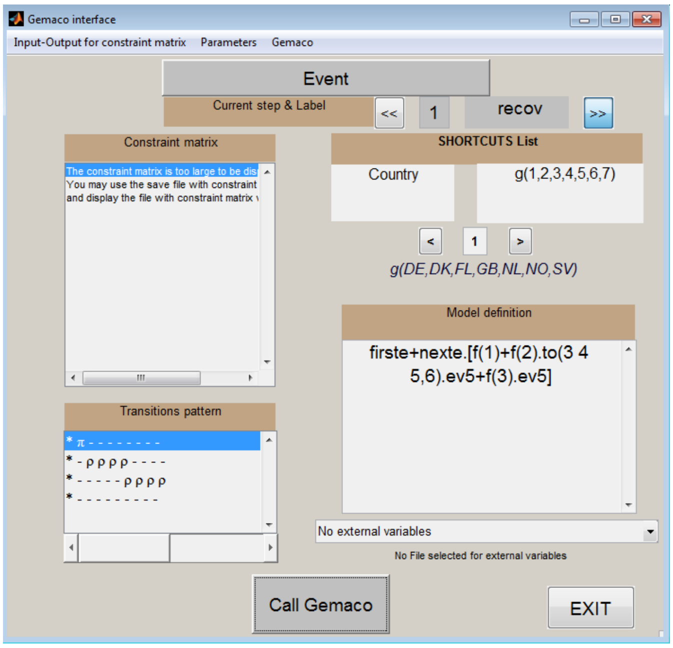

Now, we have to set the constraints for the last parameters (assignment). We want constant assignment probabilities over time, that are different depending on the cause of death (hunting vs other).

The parameters for the hunting cause of death are on rows 3 to 6, for the other causes of death on rows 7 to 10. To pool parameter from different rows, we will use the "f" shortcut and set in the same list all the parameters to be pooled: *f(3 4 5 6,7 8 9 10).*

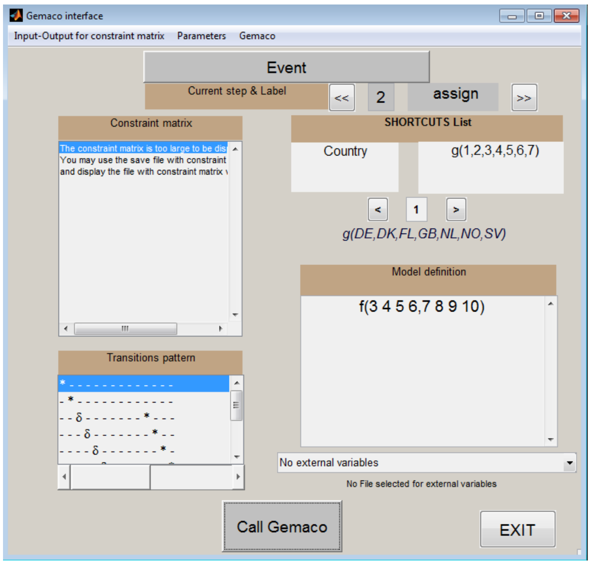

Then, click outside the sentence and then on "Call gemaco".

Now, all constraints for each matrix are defined and we exit the GEMACO interface. Then click on “EXIT” in the lower-right corner of the GEMACO interface. Now, the IVFV button is available.

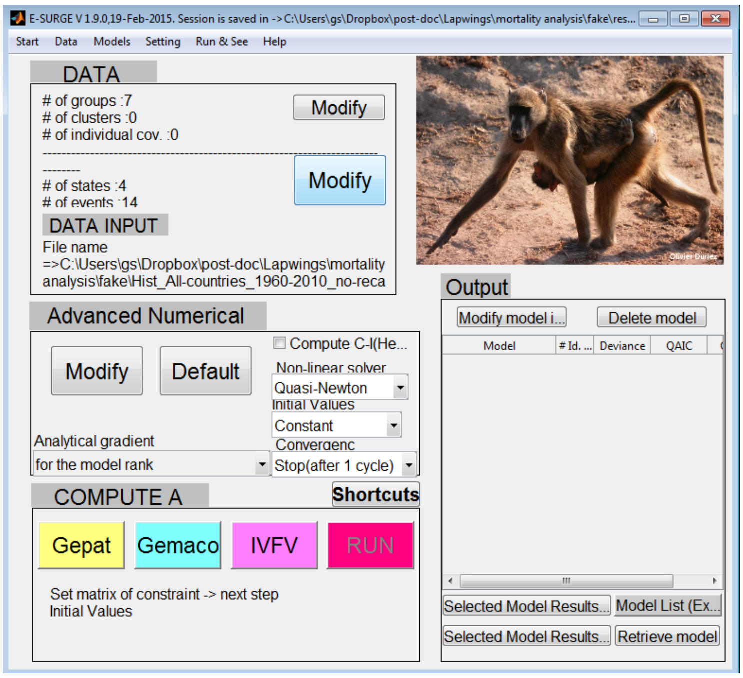

5. IVFV (Initial Value or Fixed Value for betas)

In this section we can specify initial values for the numerical calculations and fix parameter values. For our model we just need to fix 2 parameters: the capture probability during the initial capture (fixed to 1 because we have captured all individuals when they are marked); the live recapture probability (fixed to 0 because no alive reencounteres were included in the dataset). Click on the IVFV button.

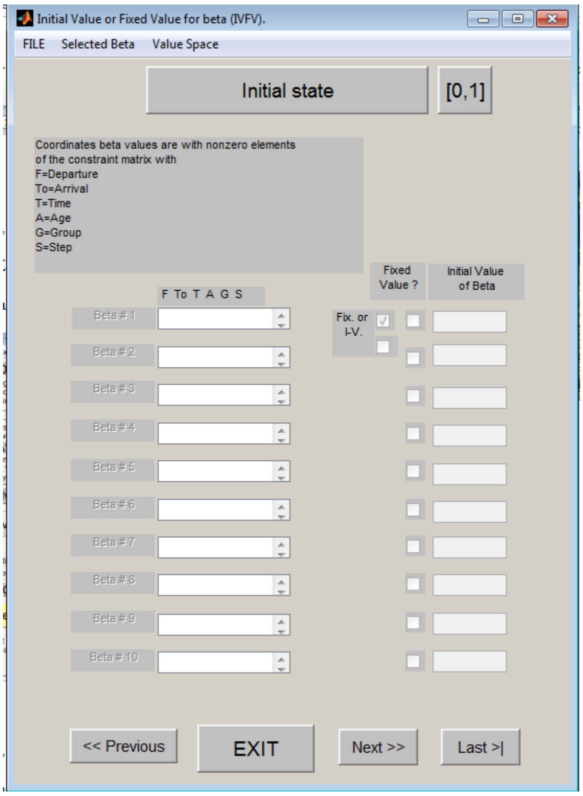

As in the other interfaces, we can access parameters for the Initial State, Transition or Event by clicking on the large button in the upper part of the interface,.

Because, we only need to fix Event parameters, click twice to reach the Event interface.

This interface shows the betas, i.e. the mathematical parameters and their “definitions”.

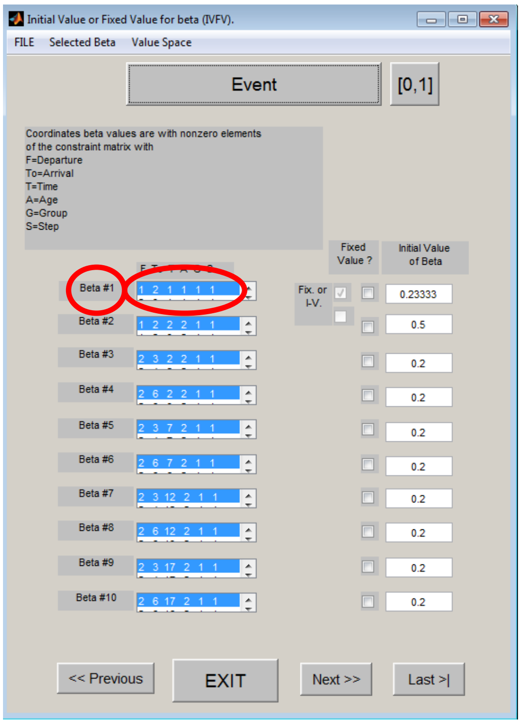

The coordinates/definition reminds which parameter is linked to beta: coordinates in the matrix (F, TO), the time, age, group and step of the parameters.

Remind that age 1 refer to the first capture of an individual and thus, age 2 refers to subsequent reencounters.

Thus beta #1 (1 2 1 1 1 1) for the Event process is the beta from step 1 (i.e. recovery step), group 1, age 1, time 1 and in column 2 and row 1. Thus, this is the initial capture probability at time 1.

Beta #2 is the capture probability [F(1).To(2)] at time 2, age 2. This is thus the recapture probability.

Beta #3 is the recovery probability (step 1) due to hunting [f(2)] in area BI [to(3)] at time 2 for age 2, group 1.

As we need to fix the initial capture probability to 1 and the live recapture probability to 0, we need to fix beta 1 and beta 2 to 1 and 0, respectively.

Select the number in front of beta 1, and change the value for “1”. Then tick the case under “Fixed Value?” to say that the parameter is set to 1 and does not need to be estimated.

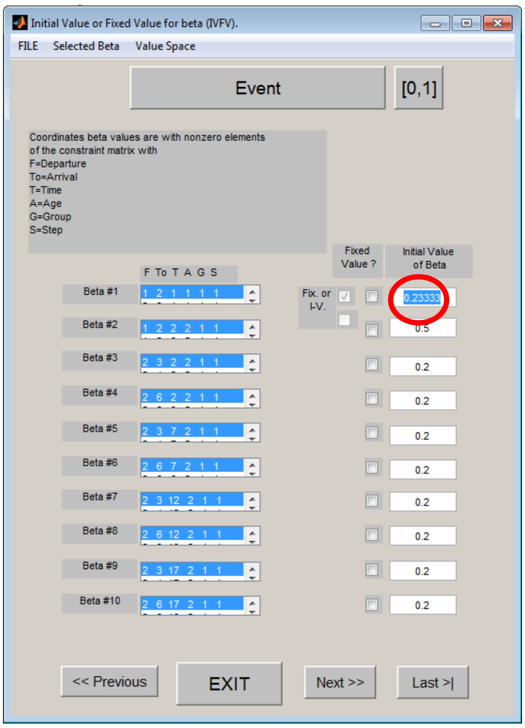

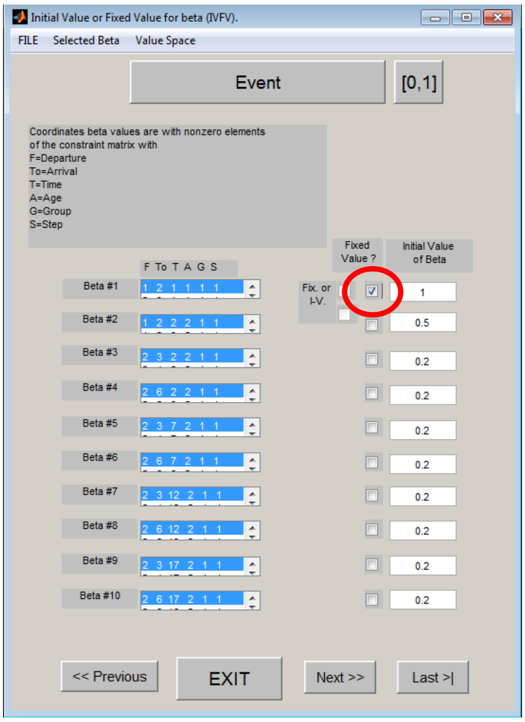

Do the same for beta 2 but with value 0

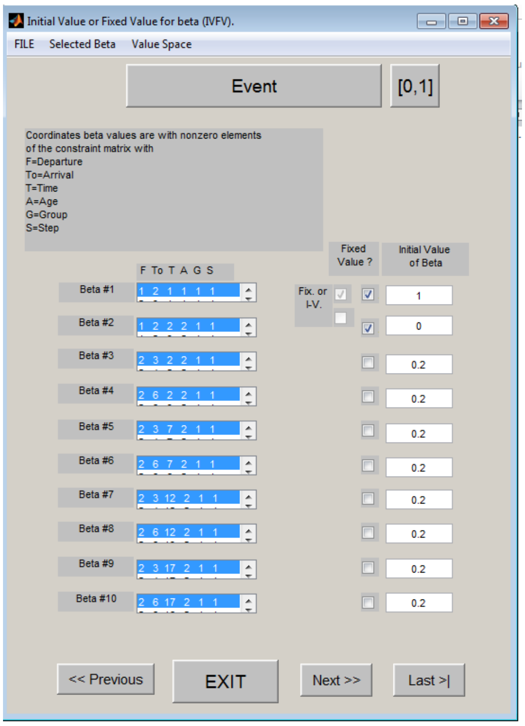

Now, we have fixed the values and can leave this interface, thus click on “Exit”. E-SURGE creates temporary files corresponding to the data which can take several minutes, depending on the overall number of parameters..

Now, the button “RUN” becomes available.

6. Run the model

Click on “Run” to run the model.

For further options (advanced numerical options, different initial values), look at the E-SURGE manual.

A web forum is also dedicated to analysis questions about capture-mark-recapture models with a special section about E-SURGE (http://www.phidot.org/forum/viewforum.php?f=15&sid=b422110643761e7e0496ead2b04bafa2).

A google group is now available and one can easily join this group (https://groups.google.com/forum/#!forum/esurge_ucare).

Feel free to send a question on this website. An answer will be posted either by members of the developing team or by experienced users.
