# Supplementary material for: Investigating Rates of Hunting and Survival in Declining European Lapwing Populations
Source: PLoS One. 2016 Sep 29;11(9):e0163850. doi: 10.1371/journal.pone.0163850 (PMC5042549; doi:10.1371/journal.pone.0163850)
Supplement: S3 File — Brief description of the model used to estimate survival and recovery probabilities while taking into account uncertainty in the recovery process. (PDF) [file pone.0163850.s003.pdf]

# S3 Multievent Seber model

This multievent Seber model is a simplified version of the multievent cause-specific mortality model. In the former model, the proportion of individuals that died due to hunting (α) is not directly estimated, but just survival and cause-specific recovery probabilities. Consequently contains the model just 3 true states, namely alive (A), newly dead (ND) and dead (D). The state process is described by the following transition matrix:

$$\begin{array}{c} \\ A \\ ND \\ D \end{array} \begin{array}{c} \begin{array}{ccc} A & ND & D \end{array} \\ \begin{bmatrix} S & 1-S & 0 \\ 0 & 0 & 1 \\ 0 & 0 & 1 \end{bmatrix} \end{array}$$

The observation states of the multievent Seber model are identical to those from the multievent cause-specific mortality model and the observation process is similar:

$$\begin{array}{c} \\ A \\ ND \\ D \end{array} \begin{array}{c} \begin{array}{cccccccccccccc} 0 & 1 & 2 & 3 & 4 & 5 & 6 & 7 & 8 & 9 & 10 & 11 & 12 & 13 \end{array} \\ \begin{bmatrix} 1 & 0 & 0 & 0 & 0 & 0 & 0 & 0 & 0 & 0 & 0 & 0 & 0 & 0 \\ 1-\sum \rho_j^i & 0 & \rho_h^{BI}\delta_h & \rho_h^{NE}\delta_h & \rho_h^{SC}\delta_h & \rho_h^{WM}\delta_h & \rho_o^{BI}\delta_o & \rho_o^{NE}\delta_o & \rho_o^{SC}\delta_o & \rho_o^{WM}\delta_o 0 & \rho_j^{BI}(1-\delta_j) & \rho_j^{NE}(1-\delta_j) & \rho_j^{SC}(1-\delta_j) & \rho_j^{WM}(1-\delta_j) \\ 1 & 0 & 0 & 0 & 0 & 0 & 0 & 0 & 0 & 0 & 0 & 0 & 0 & 0 \end{bmatrix}_t \end{array}$$

The significance of the recovery probabilities (ρ) differs from the recovery probabilities of the multievent cause-specific mortality model ($r$). Because the $\rho_j^i$ condition on death only (not on a specific cause of death), they are defined as the probability that a dead bird has died due to a specific reason and its ring is reported. Thus, $\rho_h$ is defined as the probability that a ringed lapwing died, was retrieved and its ring reported as dead due to hunting and $\rho_o$ is the probability that a ringed dead lapwing died and its ring was reported as dead due to another cause than hunting. These two recovery probabilities thus contain α.

Because we wanted to estimate kill rates specific for different ringing regions, age classes and time periods, the fitted multievent Seber model was:

$$S_{[Reg*a*y5]}, \rho_{h[Reg*a*y5]}, \rho_o, \delta_h, \delta_o$$
