# Supplementary material for: Investigating Rates of Hunting and Survival in Declining European Lapwing Populations
Source: PLoS One. 2016 Sep 29;11(9):e0163850. doi: 10.1371/journal.pone.0163850 (PMC5042549; doi:10.1371/journal.pone.0163850)
Supplement: S4 File — Tables summarizing reporting rates and crippling loss rates of Mourning doves from the literature. (PDF) [file pone.0163850.s004.pdf]

S4 Review of reporting rates and crippling loss rates of Mourning doves.

Table A: Review of reporting rates of Mourning doves from North-America.

Period	Country	Region	Reporting rate	Remark	References
1965-1966	USA		0.32	No solicitation	Tomlisom 1968
			0.66	With solicitation	
1970-1972	USA	Eastern	0.33		Reeves 1979
		Central	0.45		
1965-1975	USA	Central	0.35		Otis 2002
1995-1998	USA	Ohio	0.21 [0.14 – 0.27]		Scott et al. 2004
2003-2005	USA	Eastern	0.53		Otis et al. 2008
		Central	0.57		
		Western	0.57		
		overall	0.55		
2008-2010	USA	Western	0.50 ± 0.04	Toll-free number	Sanders & Otis 2012
			0.54 ± 0.04	Web	
		Central	0.46 ± 0.05	Toll-free number	
			0.49 ± 0.06	Web	
		Eastern	0.54 ± 0.02	Toll-free number	
			0.58 ± 0.02	Web	

Table B: Review of crippling loss values of Mourning doves from North-America.

Period	Country	Region	Crippling loss	Remark	References
1973-1975	USA	South Carolina	27-41		Haas 1977
2005-2008		Missouri	25 (range: 18 - 50)	Radiomarked	Schulz et al. 2013
2005-2011			16[1] (range: 14 - 18)	Standard	

The crippling loss (l) is the complement of the retrieval rate:

$$c = 1 - l$$

Literature Cited

Haas, G.H. (1977) Unretrieved Shooting Loss of Mourning Doves in North-Central South Carolina. *Wildlife Society Bulletin,* **5,** 123-125.

Reeves, H.M. (1979) Estimates of reporting rates for mourning dove bands. *Journal of Wildlife Management,* **43,** 36-42.

Otis, D.L. (2002) Survival Models for Harvest Management of Mourning Dove Populations. *Journal of Wildlife Management,* **66,** 1052-1063.

Otis, D.L., Schulz, J.H. & Scott, D.P. (2008) *Mourning dove (Zenaida macroura) harvest and population parameters derived from a national banding study*. US Department of the Interior, Fish and Wildlife Service, Washington, D.C., USA.

Sanders, T.A. & Otis, D.L. (2012) Mourning dove reporting probabilities for web-address versus toll-free bands. *Journal of Wildlife Management,* **76,** 480-488.

Scott, D.P., Berdeen, J.B., Otis, D.L. & Fendrick, R.L. (2004) Harvest parameters of urban and rural mourning doves in Ohio. *Journal of Wildlife Management,* **68,** 694-700.

Schulz, J.H., Bonnot, T.W., Millspaugh, J.J. & Mong, T.W. (2013) Harvest and crippling rates of mourning doves in Missouri. *Wildlife Society Bulletin,* **37,** 287-292.

Tomlinson, R.E. (1968) Reward banding to determine reporting rate of recovered mourning dove bands. *Journal of Wildlife Management,* **32,** 6-11.
