# Supplementary material for: Investigating Rates of Hunting and Survival in Declining European Lapwing Populations
Source: PLoS One. 2016 Sep 29;11(9):e0163850. doi: 10.1371/journal.pone.0163850 (PMC5042549; doi:10.1371/journal.pone.0163850)
Supplement: S5 File — (PDF) [file pone.0163850.s005.pdf]

S5 Point estimates (± SE) of several demographic parameters for lapwing populations from 1960 to 2010.

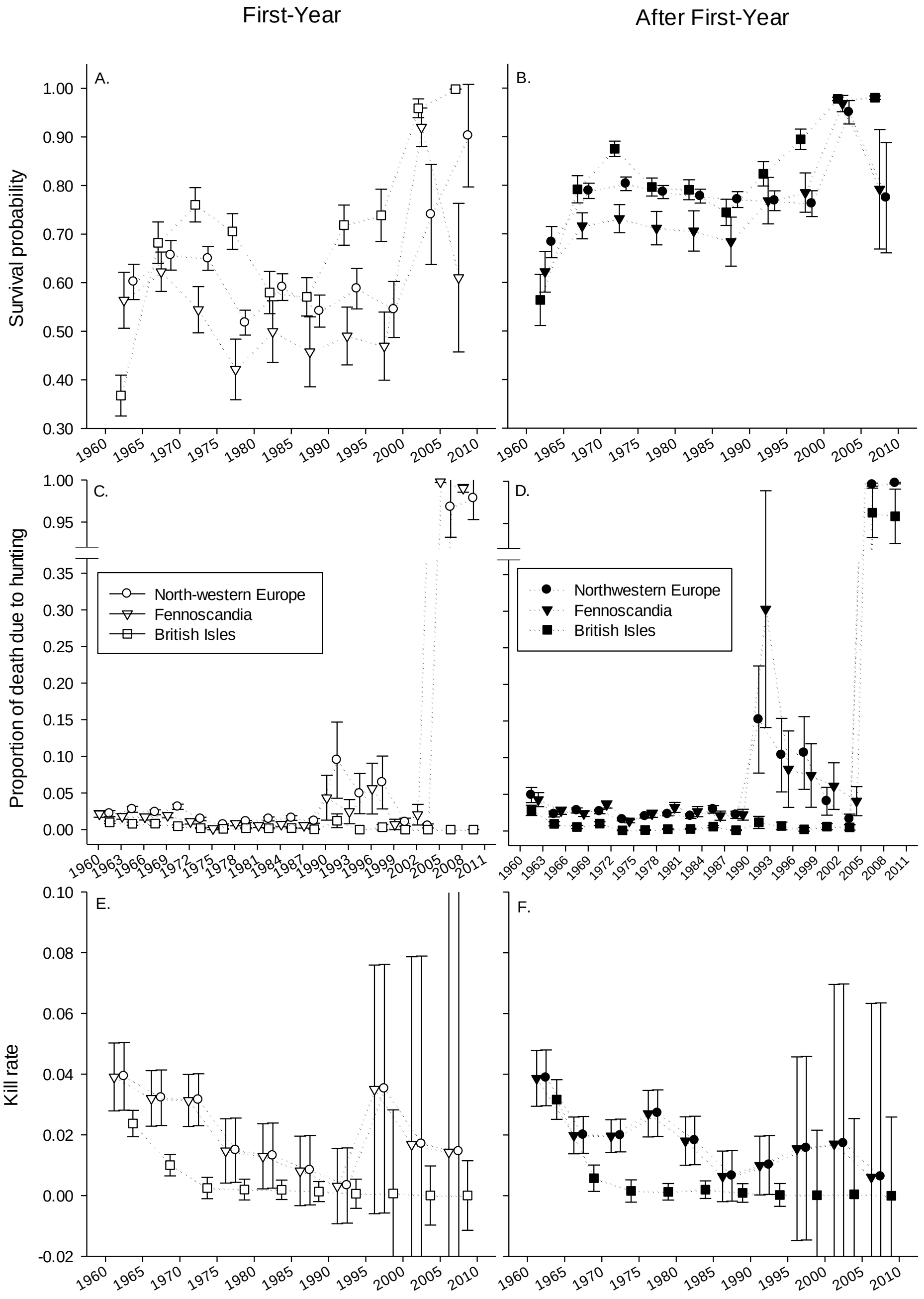

Figure A: Estimates (± SE) of annual survival, proportion of mortality due to hunting and kill rate of lapwings from 1960 to 2010 by area of ringing (circles, triangles and squares represent North-western Europe, Fennoscandia and British Isles respectively). First row: annual survival probability, second row: proportion of mortality due to hunting, third row: kill rate. Left column: first-year, Right column: after first-year.

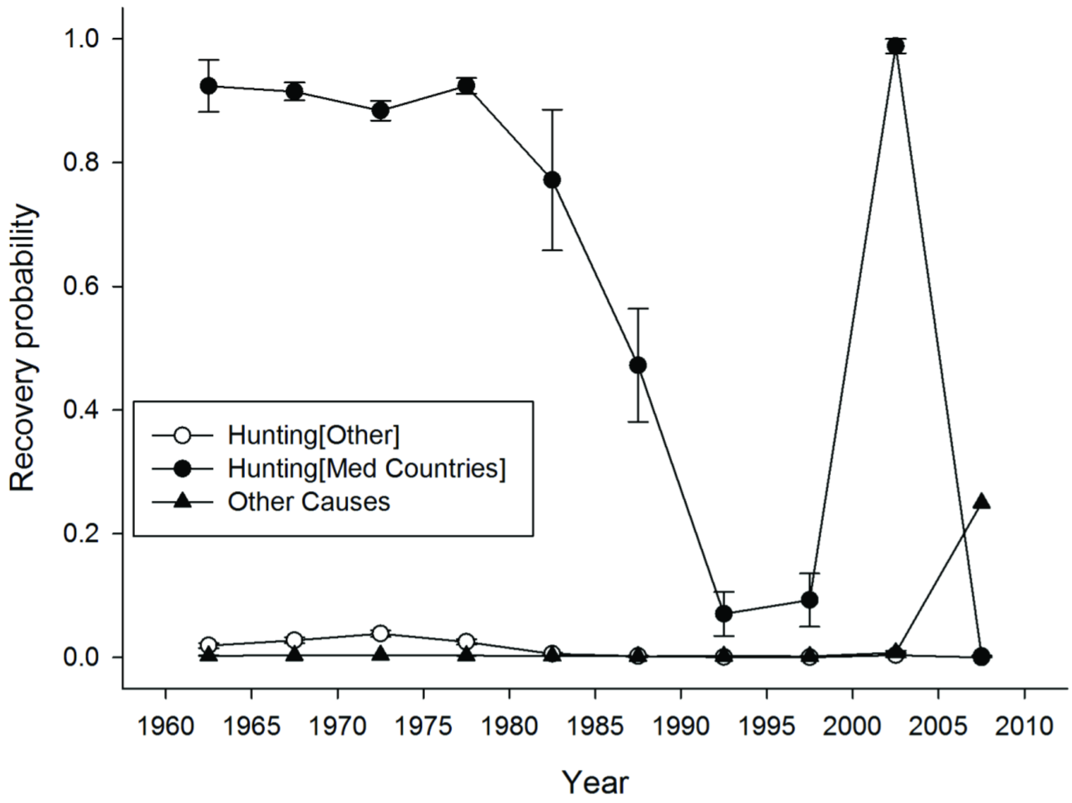

Figure B: Estimates (± SE) of annual cause-specific recovery probability of lapwings from 1960 to 2010.

Circles and triangles represent hunting and other sources of death, respectively. Full and open circles represent hunting recovery probabilities in countries around the Western Mediterranean Sea and elsewhere, respectively.
