# Supplementary material for: Investigating Rates of Hunting and Survival in Declining European Lapwing Populations
Source: PLoS One. 2016 Sep 29;11(9):e0163850. doi: 10.1371/journal.pone.0163850 (PMC5042549; doi:10.1371/journal.pone.0163850)
Supplement: S6 File — Comparison of mean and standard errors survival and proportion of death due to hunting between the multievent cause-specific mortality model and the standard cause-specific mortality model [16] where recoveries with unknown cause of death where either assigned to another cause of death (i.e. not harvested) or discarded. (PDF) [file pone.0163850.s006.pdf]

S6 On the use of recoveries with unknown source of death in the standard cause-specific mortality model.

To investigate the effect of using data with unknown cause of death using the standard cause-specific mortality model (Schaub and Pradel 2004), we compared points estimates and associated standard errors between the multievent cause-specific model and the standard cause-specific mortality model where either recoveries with unknown cause of death were assigned to the "other cause" group or where recoveries with unknown cause of death were discarded.

We found that survival estimates were similar among all models, despite some parameters were estimated at boundaries in the standard cause-specific mortality model when unknown recoveries were assigned to a specific cause of death. The precision of the estimates, reflected by their standard errors, were lower when the multievent cause-specific mortality model was used (Fig A).

The comparison of the proportion of death due to hunting between the three different settings shows that assigning recoveries with unknown cause of death to another cause of death or discarding them biases these parameters. This is likely because not all the birds recorded with unknown causes of death were actually dead due to causes different from hunting (Fig B).

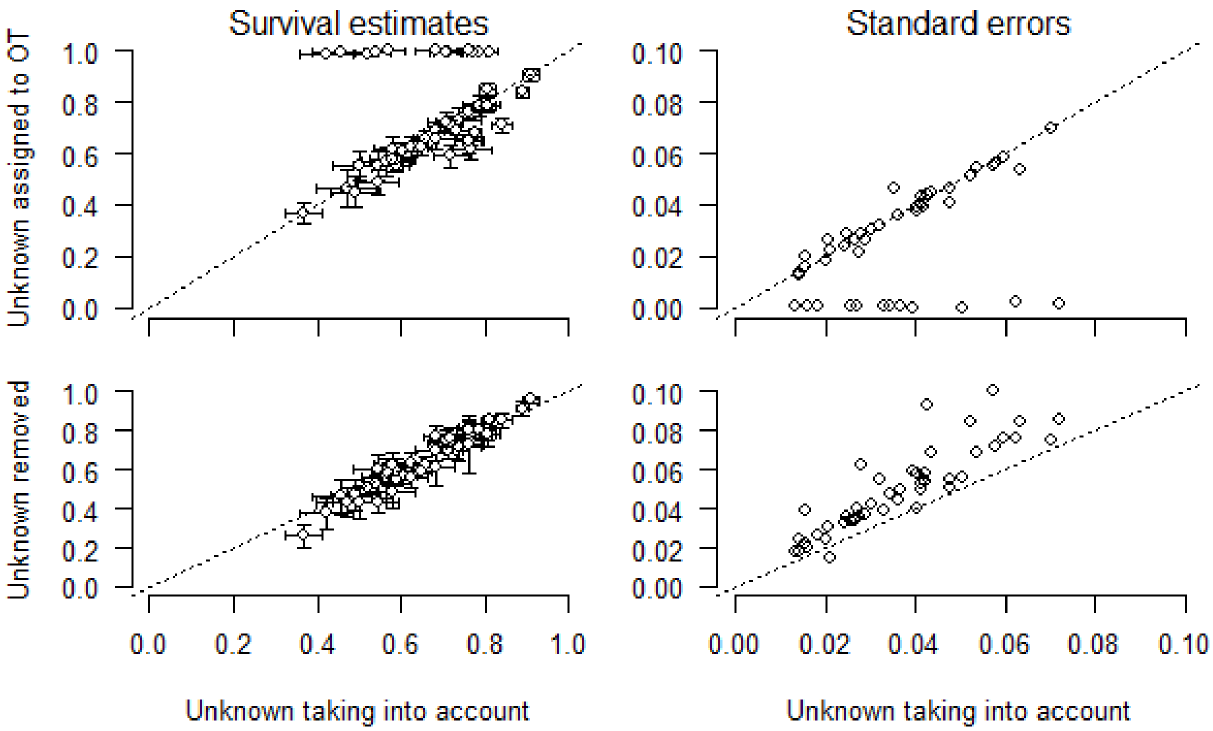

Fig A. Comparison of survival probabilities and associated standard errors between multievent cause-specific mortality model and standard cause-specific mortality model where recoveries with unknown cause of death were either assigned to other causes of death or discarded. Panels in the first row show comparison between the multievent cause-specific mortality model (x-axis) and the standard cause-specific mortality model where recoveries with unknown cause of death were assigned to other causes of death (y-axis), panels in the second row show comparison between multievent cause-specific mortality model (x-axis) and the standard cause-specific mortality model where recoveries with unknown cause of death were discarded (y-axis). The dotted line represents a y=x regression line.

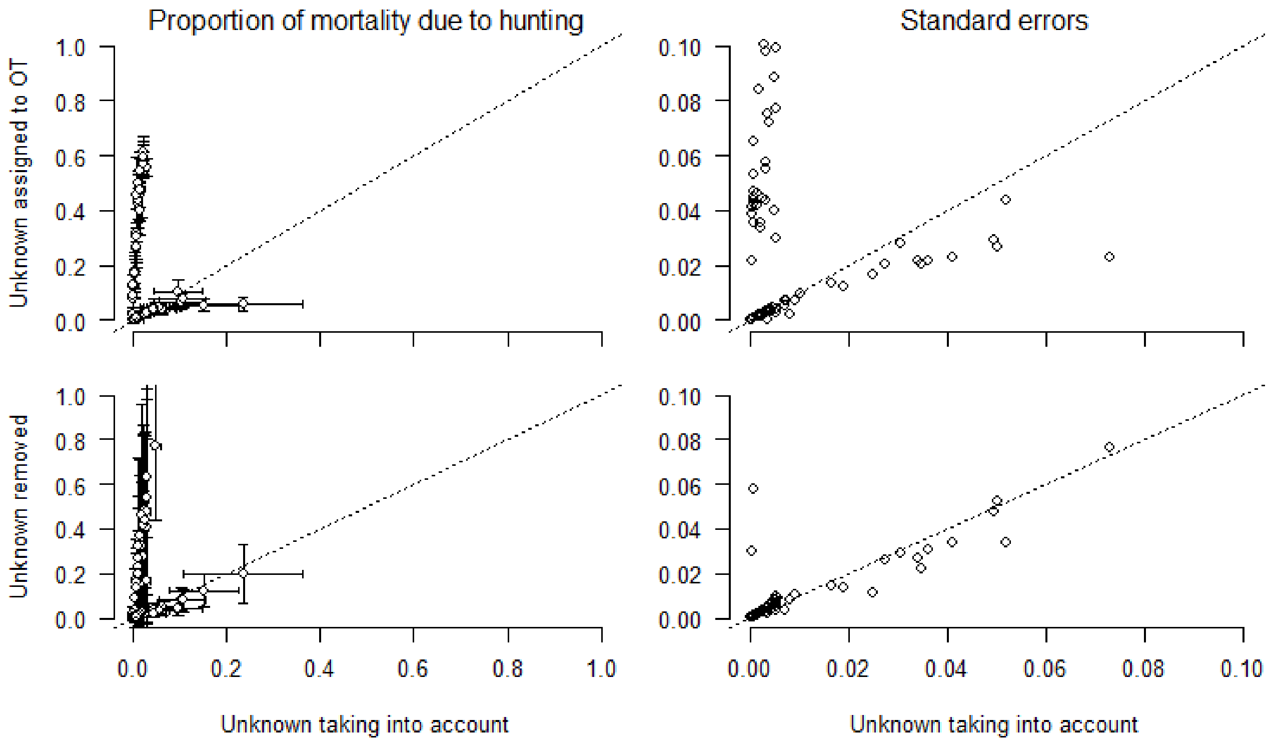

Fig B. Comparison of estimated proportions of death due to hunting and associated standard errors between the multievent cause-specific mortality model and standard cause-specific mortality model where recoveries with unknown cause of death were either assigned to other causes of death or discarded. Panels in the first row show comparison between the multievent cause-specific mortality model (x-axis) and the standard cause-specific mortality model where recoveries with unknown cause of death were assigned to other causes of death (y-axis), panels in the second row show comparison between multievent cause-specific mortality model (x-axis) and the standard cause-specific mortality model where recoveries with unknown cause of death were discarded (y-axis). The dotted line represents a y=x regression line.
