# Supplementary material for: Investigating Rates of Hunting and Survival in Declining European Lapwing Populations
Source: PLoS One. 2016 Sep 29;11(9):e0163850. doi: 10.1371/journal.pone.0163850 (PMC5042549; doi:10.1371/journal.pone.0163850)
Supplement: S7 File — (PDF) [file pone.0163850.s007.pdf]

S7 Comparison of parameter estimates obtained from the multievent cause-specific mortality model and a multievent Seber model.

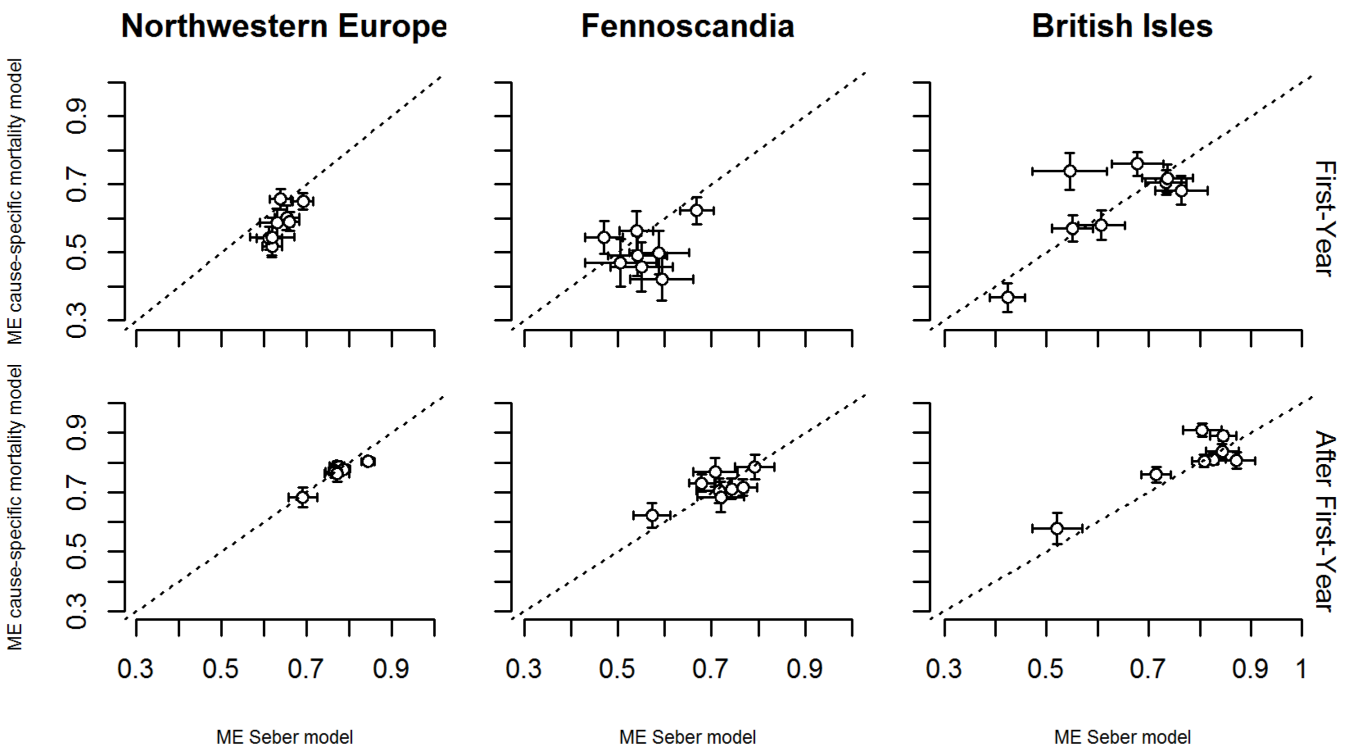

Fig A: Comparison of survival probabilities (± SE) obtained from a multievent Seber model (x-axis) and the multievent cause-specific mortality model (y-axis). Panels in the first row show results for FY individuals, panels in the second row the results for AFY individuals. Panels in different columns refer to the origin of the lapwings. The dotted line represents a y=x regression line.

Table A: Mean and SE (in parentheses) of survival (S) and recovery (r) probabilities of lapwings estimated with the multievent recovery Seber model used for the computation of the kill rates. FY: first-year birds; AFY: after first-year birds.

Period	Northwestern Europe				Fennoscandia				British Isles			
	Survival *(S)*		Recovery (r_h)		Survival *(S)*		Recovery (r_h)		Survival *(S)*		Recovery (r_h)	
	FY	AFY	FY	AFY	FY	AFY	FY	AFY	FY	AFY	FY	AFY
1960-65	0.58	0.66	0.027	0.035	0.55	0.62	0.023	0.028	0.35	0.55	0.010	0.019
	(0.04)	(0.04)	(0.004)	(0.006)	(0.07)	(0.05)	(0.004)	(0.006)	(0.04)	(0.05)	(0.001)	(0.004)
1965-70	0.62	0.82	0.022	0.038	0.67	0.73	0.026	0.020	0.70	0.82	0.009	0.009
	(0.03)	(0.02)	(0.003)	(0.005)	(0.04)	(0.03)	(0.005)	(0.003)	(0.04)	(0.03)	(0.002)	(0.002)
1970-75	0.63	0.78	0.023	0.021	0.46	0.67	0.015	0.016	0.70	0.87	0.002	0.004
	(0.03)	(0.02)	(0.003)	(0.002)	(0.05)	(0.03)	(0.003)	(0.002)	(0.04)	(0.02)	(0.001)	(0.001)
1975-80	0.48	0.79	0.008	0.026	0.39	0.74	0.006	0.028	0.72	0.82	0.002	0.002
	(0.02)	(0.01)	(0.001)	(0.002)	(0.07)	(0.03)	(0.002)	(0.006)	(0.04)	(0.02)	(0.001)	(0.001)
1980-85	0.59	0.79	0.012	0.022	0.49	0.73	0.007	0.018	0.58	0.82	0.001	0.003
	(0.03)	(0.01)	(0.001)	(0.002)	(0.07)	(0.04)	(0.002)	(0.004)	(0.04)	(0.02)	(0.000)	(0.001)
1985-90	0.53	0.76	0.007	0.012	0.48	0.68	0.004	0.005	0.57	0.74	0.001	0.001
	(0.03)	(0.02)	(0.001)	(0.001)	(0.07)	(0.05)	(0.001)	(0.002)	(0.04)	(0.03)	(0.000)	(0.000)
1990-95	0.57	0.77	0.006	0.011	0.45	0.76	0.002	0.011	0.69	0.82	0.000	0.001
	(0.05)	(0.02)	(0.001)	(0.001)	(0.06)	(0.05)	(0.001)	(0.003)	(0.04)	(0.02)	(0.000)	(0.000)
1995-00	0.48	0.75	0.004	0.010	0.47	0.78	0.005	0.005	0.69	0.88	0.000	0.000
	(0.06)	(0.02)	(0.001)	(0.002)	(0.07)	(0.04)	(0.001)	(0.002)	(0.06)	(0.03)	(0.000)	(0.000)
2000-05	0.58	0.90	0.004	0.014	0.86	0.95	0.009	0.027	0.93	0.99	0.000	0.004
	(0.08)	(0.03)	(0.001)	(0.004)	(0.05)	(0.02)	(0.005)	(0.010)	(0.02)	(0.00)	(0.000)	(0.002)
2005-10	0.29	0.66	0.000	0.003	0.67	0.89	0.003	0.004	0.65	0.94	0.000	0.000
	(0.10)	(0.08)	(0.000)	(0.001)	(0.14)	(0.05)	(0.002)	(0.002)	(0.09)	(0.02)	(0.000)	(0.000)

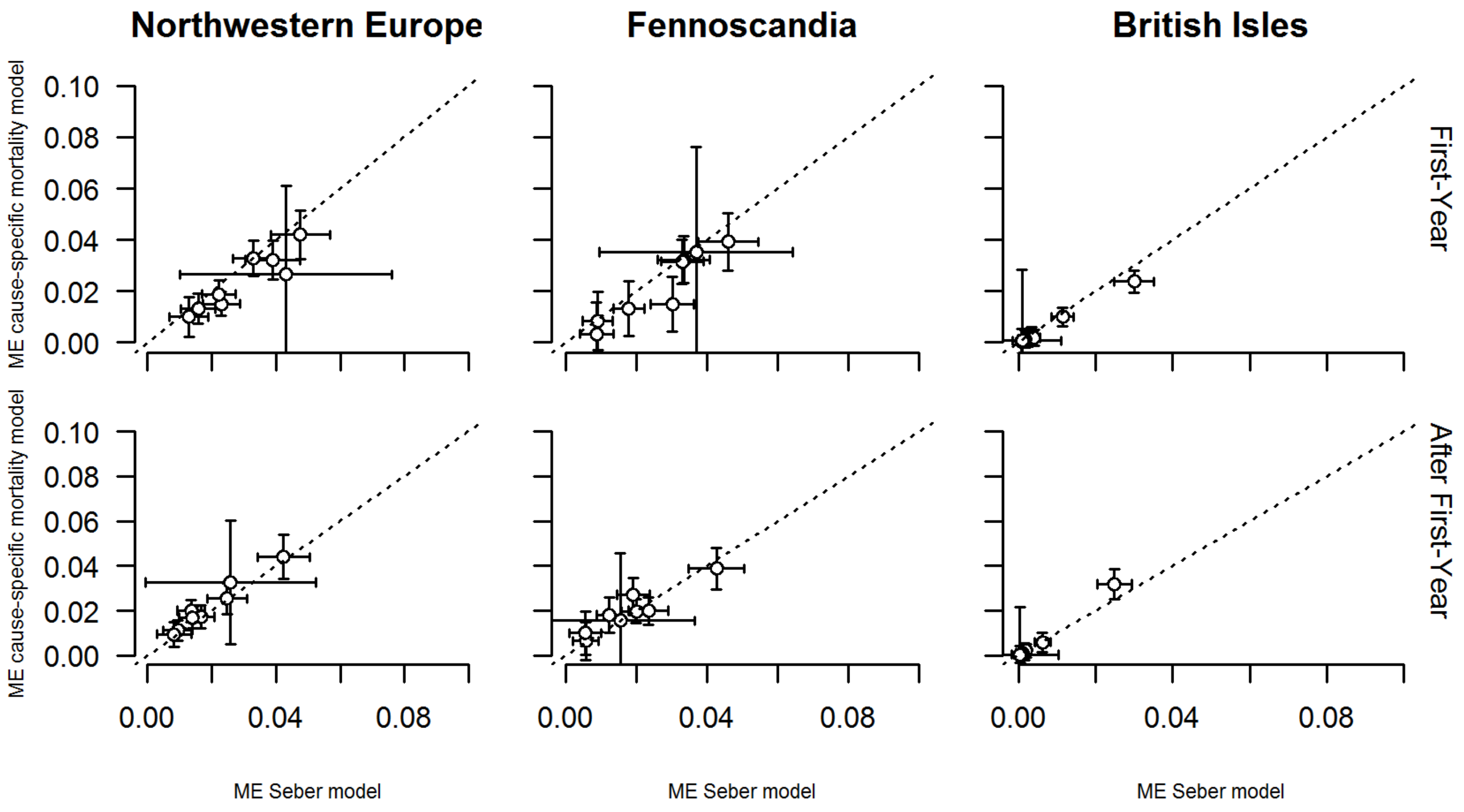

Fig B: Comparison of kill rates (± SE) estimated from a multievent Seber model (x-axis) and the multievent cause-specific mortality model (y-axis). Panels in the first row show results for FY individuals, panels in the second row the results for AFY individuals. Panels in different columns refer to the origin of the lapwings. The dotted line represents a y=x regression line.
