# Supplementary material for: Investigating Rates of Hunting and Survival in Declining European Lapwing Populations
Source: PLoS One. 2016 Sep 29;11(9):e0163850. doi: 10.1371/journal.pone.0163850 (PMC5042549; doi:10.1371/journal.pone.0163850)
Supplement: S8 File — (PDF) [file pone.0163850.s008.pdf]

S8 Comparison of kill rates computed from different values of reporting and retrieval rates.

Table A: Mean kill rate over the period 1960-2010 by age and area of ringing derived from different values of reporting and retrieval rates. FY: first-year birds, AFY: after-first year birds, NE: Northern Europe, SC: Fennoscandia, BI: British Islands. Note that the values of reporting and retrieval rates used in the paper were λ = 0.35 (1960-1990), λ = 0.10 (1990-2010) and c = 0.75.

			Retrieval rate (*c*)														
			0.9			0.8			0.7			0.6			0.5		
			NE	SC	BI	NE	SC	BI	NE	SC	BI	NE	SC	BI	NE	SC	BI
Reporting rate (λ)	0.1	FY	0.053	0.047	0.012	0.059	0.053	0.014	0.068	0.061	0.016	0.079	0.071	0.018	0.095	0.085	0.022
		AFY	0.049	0.045	0.013	0.055	0.050	0.015	0.062	0.057	0.017	0.073	0.067	0.020	0.087	0.080	0.024
	0.2	FY	0.026	0.024	0.006	0.030	0.027	0.007	0.034	0.030	0.008	0.039	0.036	0.009	0.047	0.043	0.011
		AFY	0.024	0.022	0.007	0.027	0.025	0.007	0.031	0.029	0.009	0.036	0.034	0.010	0.044	0.040	0.012
	0.3	FY	0.018	0.016	0.004	0.020	0.018	0.005	0.023	0.020	0.005	0.026	0.024	0.006	0.032	0.028	0.007
		AFY	0.016	0.015	0.004	0.018	0.017	0.005	0.021	0.019	0.006	0.024	0.022	0.007	0.029	0.027	0.008
	0.4	FY	0.013	0.012	0.003	0.015	0.013	0.003	0.017	0.015	0.004	0.020	0.018	0.005	0.024	0.021	0.006
		AFY	0.012	0.011	0.003	0.014	0.013	0.004	0.016	0.014	0.004	0.018	0.017	0.005	0.022	0.020	0.006
	0.5	FY	0.011	0.009	0.002	0.012	0.011	0.003	0.014	0.012	0.003	0.016	0.014	0.004	0.019	0.017	0.004
		AFY	0.010	0.009	0.003	0.011	0.010	0.003	0.012	0.011	0.003	0.015	0.013	0.004	0.017	0.016	0.005
